# Insights into the metastatic bone marrow niche gained from fibronectin and β1 integrin transgenic mice

**DOI:** 10.1016/j.neo.2024.101058

**Published:** 2024-10-15

**Authors:** Franziska Wirth, Caren Zoeller, Alexander Lubosch, Jutta Schroeder-Braunstein, Guido Wabnitz, Inaam A. Nakchbandi

**Affiliations:** a Institute of Immunology, Heidelberg University, 69120, Heidelberg, Germany; b Max-Planck Institute for Biochemistry, 82152, Martinsried, Germany; c Max-Planck Institute for Medical Research, 69120, Heidelberg, Germany

**Keywords:** Extracellular matrix, Fibronectin, β1 integrin, Bone marrow, Breast cancer, Metastatic niche, Pre-metastatic niche, Hematopoietic stem cell niche, Stem cell niche, Homing, Hematopoiesis, Myelopoiesis

## Abstract

Tumor cells can migrate from a primary cancer and form metastases by localizing to niches within other organs including the bone marrow, where tumor cells may exploit the hematopoietic stem cell niche. The precise composition of the premetastatic and the hematopoietic niches and the degree of overlap between them remain elusive. Because the extracellular matrix protein fibronectin is expressed in the pre-metastatic lung microenvironment, we evaluated the implications of its loss, as well as those of loss of its primary receptor subunit, β1 integrin, in various bone marrow cell types both in breast cancer bone metastasis and hematopoiesis.

Using eight transgenic mouse models, we established that fibronectin production by osterix-expressing marrow cells, or β1 integrin expression (on vav, mx, or leptin receptor expressing cells), affects MDA-MB-231 breast cancer cell numbers in the bone marrow. Additionally, we identified stromal subpopulations that modulate transmigration through blood vessel walls. Not the number of tumor cells, but rather the changes in the microenvironment dictated whether the tumor progresses. Furthermore, hematopoiesis, particularly myelopoiesis, was affected in some of the models showing changes in tumor homing.

In conclusion, there is partial overlap between the pre-metastatic and the hematopoietic niches in the bone marrow. Moreover, we have delineated a cascade starting with fibronectin secreted by pre-osteoblastic cells, which potentially acts on β1 integrin in specific stromal cell subsets, thereby inhibiting the formation of new breast cancer lesions in the bone marrow. This work therefore sheds light on the role of various stromal cell subpopulations that influence tumor behavior and affect hematopoiesis.

## Highlights

• Changes in stromal cells mostly, but not always, impact both the pre-metastatic niche for breast cancer and the hematopoietic stem cell niche
• Fibronectin production or β1 integrin expression in specific stromal subpopulations affect the number of tumor cells, the development of metastatic lesions and hematopoiesis differently
• Fibronectin and β1 integrin influence hematopoiesis, especially early myelopoiesis

## Graphical abstract

**Figure:**
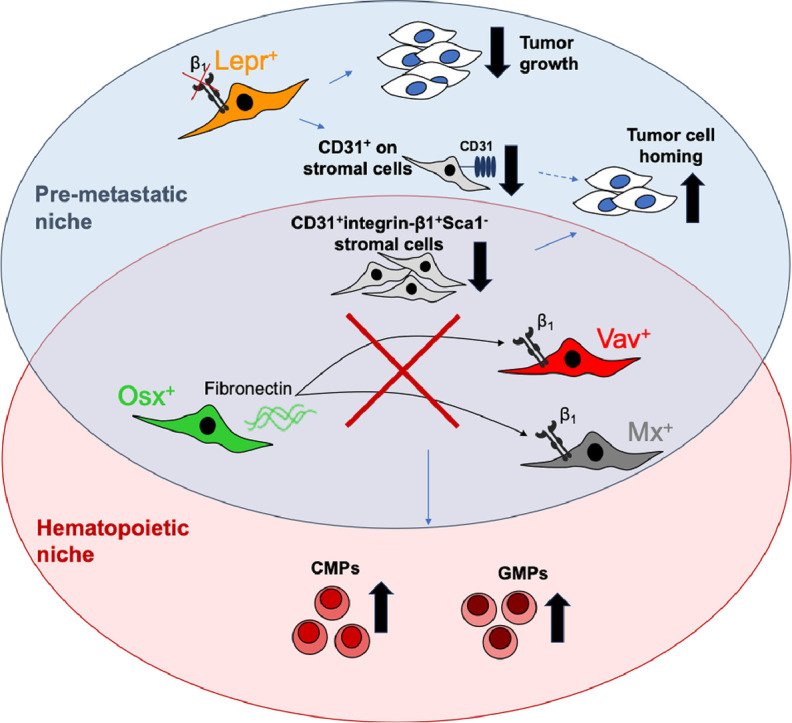

## Introduction

Metastatic lesions reflect the spread of tumor cells from their original site to new organs, where they localize in the so-called metastatic niche and generally forebode a poor prognosis [1,2]. Despite many advances, the mechanisms underlying recruitment and integration of tumor cells within these niches, in particular in the bone marrow, remain elusive [3]. Therefore much effort has been spent on characterizing these niches. Indeed, fibronectin, an extracellular matrix protein produced by almost all mammalian cells, was found to be enriched in future sites of lung metastatic lesions [1,4]. Since it is a chemoattractant, it facilitates the recruitment of cancer cells to fibronectin-rich niches [5,4]. Additionally, fibronectin supports cell proliferation [6], differentiation and survival [7,8], and inhibits apoptosis of various cells including breast and prostate cancer [[6], [9]]. Consequently, high fibronectin expression is associated with poor outcomes in both breast and prostate cancer patients [6].

Fibronectin exerts its functions by binding to cell surface receptors called integrins. These receptors usually consist of an α and a β subunit. The β1 subunit is of particular importance because it can pair with 11 different α subunits, some of which bind to fibronectin, such as α4β1 integrin [10,11]. Interestingly, in addition to fibronectin, this integrin pair is expressed in pre-metastatic sites in the lung [1,3].

The bone marrow offers a unique environment where in particular breast and prostate cancer types can thrive [12]. Although the composition of the bone marrow pre-metastatic niche remains largely unknown, a causal relationship between bone marrow stromal cells and cancer cell prevalence was found. Injecting breast and prostate cancer cells into the blood stream after decreasing the number of bone marrow stromal cells resulted in more tumor cells detected in the bone marrow, while increasing the number of stromal cells by injecting them led to a lower number of cancer cells in the bone marrow. This suggests that specific cell populations might influence cancer cell migration into the bone marrow [13]. In particular, a small subpopulation identified as CD31^+^Sca-1^+^CD146^+^CD44^-^ showed an inverse correlation with the number of cancer cells detected in the bone marrow in mice [13]. This population, however, differed from the best predictive population of stromal cells in the bone marrow of patients with either breast or prostate cancer in whom tumor cells were detected in the bone marrow. Even though this discrepancy could result from the difference between mice and humans or from variations in cancer types, the question for us remained whether we could better characterize the stromal population that affects the number of breast cancer cells in the marrow and consequently the development of bone metastatic lesions [13].

Similarly to tumor cells in the bone marrow, hematopoietic stem cells typically reside in poorly defined niches, they divide and differentiate based on need, and mostly maintain a quiescent or resting state in response to signals from their microenvironment [14]. Even though it might be beneficial for the patients if tumor cells remained under the influence of dormancy-inducing signals and hence avoid growth of metastatic lesions, such signals also make tumor cells resistant to chemotherapy that targets proliferating cells [15,16]. It still remains unclear to what extent the pre-metastatic and the hematopoietic niches overlap.

Our knowledge on the role of fibronectin, a major matrix protein produced by stromal cells, in hematopoiesis is also limited, but fibronectin supports proliferation and survival of hematopoietic stem cells [[17], [18], [19], [20], [21]]. An additional possible function of fibronectin is retention of stem cells in the niche through integrin α4β1 [22,23].

Taken together, our limited knowledge of the composition of the pre-metastatic bone marrow niche and/or hematopoietic stem cell niche restricts our understanding of the mechanisms that regulate homing of tumor cells to the niche and the details of the interaction of tumor cells with cells of the hematopoietic stem cell niche. To shed some light on these intricate processes, we mated a total of 8 transgenic mouse lines in 16 combinations to conditionally delete fibronectin or β1 integrin using 5 promoters active in the bone marrow. We then examined the ability of breast cancer cells to lodge in the bone marrow and embarked on the identification of a bone marrow stromal subpopulation that could modulate breast cancer behavior and/or affect hematopoiesis. The goal is to characterize the interaction between the tumor cells and hematopoietic stem cells with their niches, because a deeper understanding of the niche is paramount to develop new therapeutic strategies.

## Materials and methods

### Mice

Five transgenic mouse models possessing a promoter attached to cre recombinase were used. These were: vav-cre expressed in hematopoietic and bone marrow stromal cells (Jax#008610), mx-cre, also expressed in a variety of cells. The osterix promoter attached to cre is active in the absence of doxycycline in preosteoblasts and stromal cells able to differentiate to chondrocytes or adipocytes. The nestin promoter attached to cre is active in neural tissue but has been found to be expressed in bone marrow cells (Jax#003771), and leptin receptor-cre was also reported to be expressed in stromal cells (Jax#008320) [[27], [28], [29], [30], [31], [32], [63]].

In order to determine which cells are affected the tdTomato reporter mice were used (JAX #007909). In these mice, a floxed STOP-codon prevents transcription of the red fluorescent protein tdTomato. If, in a cell, a promoter attached to cre recombinase is activated, the recombinase will be expressed. Cre then removes the floxed STOP codon from the tdTomato gene. Consequently, cells in which the promoter of interest is active will produce tdTomato that can be detected on histology sections or by flow cytometry. The aim was to delete either fibronectin (FN^fl/fl^) or β1 integrin (β1^fl/fl^) in stromal cells using the cre-loxP system, because both systemic knockouts lead to embryonic lethality [35,36]. The two genes need to be floxed on both alleles (homozygous). CD1 nude mice (CD1-*Foxn1nu*) were used.

At the time of euthanasia, EDTA-blood was collected and red blood cells (RBCs) as well as hemoglobin and platelets were evaluated using an automated system (CELL-DYN Emerald). The white blood cell number (WBC) was confirmed by using 2 μl blood added to 40μl WBC-counting solution and incubated for 30 minutes at RT. This solution ensures lysis of RBC and staining of nuclei with crystal violet. Using a Neubauer chamber WBCs were counted.

### Experimental procedures in mice

All animal work was conducted according to relevant national and international guidelines, and all procedures were approved by the responsible regulatory body (The animal protection agency of the state of Baden-Wuerttemberg in Germany) under the numbers T-49/18, T-44/20, G-214/15, G-275/15, G-264/19, G-292/19, G-277/20, G-37/21.

### Treatments

Parathyroid hormone (human PTH) (MPI of Biochemistry, Martinsried) 400 ng/g for 4 days injected subcutaneously (s.c.) and zoledronic acid (ZA) (Hexal) 100 ng/g on days 1 and 3 was administered intraperitoneally (i.p.) in female mice 4-5 weeks in age. To induce cre-expression by the mx promoter, 250 µg polyinosinic-polycytidylic acid (pIpC) (Sigma-Aldrich) was injected i.p. three times over one week as described [32,50].

Doxycycline hydrochloride (1A Pharma) was added to drinking water to suppress the osterix promoter during embryonal development. 200 μg/ml doxycycline hydrochloride in 5% sucrose solution was used instead of drinking water in pregnant females starting on presumed embryonal day E13.5, and changed every 3 days until the pups were weaned [46].

### Intracardial injection of cancer cells

The breast cancer cells (100 μl containing 10^5^ cells of MDA-MB-231-B/luc^+^ which carry a luciferase-expressing plasmid and preferentially home to the bone) [39] were injected in the left ventricle of 4-5 week-old female mice using a 30 G needle, after anesthesia consisting of midazolam 10 µg/g (Hameln), medetomidin 1 µg/g (Alfavet) und fentanyl 0.1 µg/g (Piramal) in 0.9 % NaCl. After the procedure, an antidot consisting of atipamezole 2 μg/g (Alfavet), flumazenil (Fresenius Kabi) 0.412 µg/g, 1.2 µg/g naloxone (Hameln) in 0.9% NaCl was administered subcutaneously [64].

### Bioluminescence imaging

Growth of metastatic lesions was monitored using Xenogen IVIS-100 (Caliper Life Sciences) starting 2 weeks after injection on a weekly basis up to 10 weeks by bioluminescence imaging under isoflurane (Fresenius-Kabi) anesthesia 5 minutes after injecting luciferin 150 mg/kg (Swiss Lumix SL30101). Measurements were performed in both dorsal and ventral positions and allowed quantification in relative light units (RLU) using the software “live imaging” (version 2.5, Xenogeny). The measurement duration was one to five minutes depending on the experimental time point and the associated change in lesion size [[6], [40]].

### Cell culture of tumor cells

MDA-MB-231-B/luc^+^ were cultured in DMEM (Thermo Fisher) +10% FCS (PAN Biotech) +800 µg/ml Geneticin (Carl Roth)+1% penicillin/streptomycin (P/S)(Gibco). Cells were tested regularly for contamination with mycoplasma.

### Isolation and culture of bone marrow stromal cells

Bones were isolated under sterile conditions and the ends of the bone were cut with a pair of scissors followed by flushing of the bone marrow with D-PBS using a G27 needle. The solution was then centrifuged at 500xg for 5 minutes. Erythrocytes were lysed using 1 ml ACK-lysis buffer (ammonium chloride 8.5g/L +potassium hydrogen carbonate 1g/L + 0.1 M EDTA) for 1 minute. This was followed by adding 2 ml D-PBS and repeat centrifugation. Immune cells were depleted using 50 μl magnetic Dynabeads protein G (Invitrogen) for 10^7^ bone marrow cells. Beads were washed and incubated with 25 μl unconjugated anti-mouse CD45 (clone 30-F11, 103104, Biolegend) antibody for 30 min at RT on a gently rotating plate set at 500rpm. After removal of the uncoupled antibody through washing, the cells were incubated with the beads, again for 30 min at RT with gentle shaking in 2 ml vials. Beads were then removed and the procedure repeated twice to ensure complete removal of CD45^+^ cells. Successful depletion was confirmed in the remaining CD45^-^ stromal cells by flow cytometry.

To obtain conditioned media, 10^6^ stromal cells in 200 μl αMEM (Gibco) without additives were cultured in wells from 48-well plates for 24 hours. Collected media were then centrifuged at 500xg for 5 min and supernatant used directly or frozen at 80°C.

### Cocultures of stromal cells and HSPCs

After depletion of bone marrow cells of CD45^+^ immune cells, stromal cells were labelled using Tag-it violet, washed and cultured in α-MEM+2.5% FCS+1% P/S. When using fibronectin conditional knockout cells, the FCS used was depleted of fibronectin as described in order to avoid the chemoattractant effects of fibronectin [[32], [65]]. 5 × 10^5^ cells were resuspended in 50 μl and added to round bottom wells from a 96 well plate. After sorting the HSPCs (Lineage^-^ckit^+^Sca-1^+^), these were added at 5 × 10^4^ in 50 μl medium to each well with stromal cells and cultured. 24 hours later cell dissociation buffer was applied and cells stained for HSPCs and the various progenitors (Fig. 10A uses stromal cells from transgenic mice and Supplementary Fig. 9A uses HSPCs from transgenic mice).

### Migration assay

Inserts from Corning® FluoroBlok™ transwell system with a pore size of 3 μm in a 24-well plate were used to create a two-compartment system. 1.5 × 10^6^ isolated stromal cells (in the case of CD31: 0.7 × 10^6^ sorted CD45^-^Ter119^-^CD31^+^ cells) were added in 200 μl αMEM+2.5%FCS in the insert for 24 hours before transferring the insert to a new well that contained 700 μl DMEM+10% FCS. The same number of tumor cells prelabelled with CFSE (5 μM CFSE solution for 20 min at 37°C) was added in 50 μl (for a total volume of 950 μl). Migration was analyzed every 30 min using a fluorophore plate reader (Spark, Tecan) (Fig. 6A).

### Isolation of spleen and liver cells

Spleen and liver were manually cut to small pieces. The spleen was suspended in D-PBS and pressed through a 40 μm filter. The filter was then washed with more D-PBS. The liver pieces were incubated with DMEM + 0.05 % DNAse + 1 mg/ml collagenase for 1 hour at 37°C and then filtered. The filter was then washed with more D-PBS. After centrifugation at 500xg for 5 min, the red blood cells were lysed using ACK-lysis-buffer, the cells were washed and stained for HSPCs.

### Flow cytometry

Cells (up to 10^6^) were incubated with 50 μl staining mix containing a live-dead dye as well as the antibodies in FACS Buffer (D-PBS + 1% FCS + penicillin/streptomycin). If more cells were used (10^6^-10^7^), then more mix was also applied (100 μl). The following panels were used: Detection of tdTomato and β1 integrin staining (Method is shown in Fig. 2A-B), stromal cell characterization, adhesion molecules, HSCs and MPPs (Supplementary Fig. 12B), HSPCs (Supplementary Fig. 12C). Analysis was performed using the FlowJo software from BD.

### Cell sorting

HSPCs were sorted based on the HSPC panel to isolate Lin^-^CD127^-^c-kit^+^Sca-1^+^ cells (Supplementary Fig. 12C, sorted panel is marked as VIII and is surrounded by a red frame). First, the flushed bone marrow was subjected to ACK-lysis in order to remove the red blood cells. After washing, A mix of lineage biotin-coupled antibodies: CD5, CD45R/B220, CD11b, Ly-6G/Ly-6C (Gr1) und Ter119 was added to 10^7^ cells for 30 minutes at 4°. After washing, 30 µl MagSi-STA 3.0L beads were added in a volume of 100 μl. Incubation for 15 min on ice was followed by washing with 1 ml D-PBS and centrifugation at 500xg to remove the unbound antibodies. The beads are magnetic and carry streptavidin, which binds to biotin on the antibodies, which in turn bind to the lineage marker expressing cells. Through the use of a magnet, the beads and cells are removed. The solution, which now only contains lineage negative (lin^-^) cells is then stained for HSPCs with 100 μl FACS Buffer containing Zombie Red, lineage-marker-Streptavidin-P.O., CD127/PE-Cy7, c-Kit/PerCP-Cy5.5, Sca-1/FITC. The gating strategy is shown in Supplementary Fig. 12C. HSCs were sorted based on the panel in Supplementary Fig. 8B (Lin^-^c-kit^+^Sca-1^+^CD34^-^CD135^-^CD48^-^CD150^+^) using the following antibodies: Zombie NIR, lineage-marker-Streptavidin-P.O., CD127/PE-Cy7, c-Kit/PerCP-Cy5.5, Sca-1/FITC, CD34/PE, CD135/BV421, CD48/APC, CD150/PE-Cy7. To isolate the cells that express cre recombinase, we took advantage of the tdTomato reporter mice and stained for live-dead, CD45 and Ter119, as well as β1 integrin. The experimental strategy is shown in Fig. 2a-b. LSRII or BD LSR Fortessa X-20 were used (BD Bioscience).

Cells were always sorted for high purity. The cells were collected in αMEM+10%FCS.

### Stem cell differentiation *in vitro*

HSCs were sorted, resuspended in αMEM+2% FCS. This was mixed with 10x of the methylcellulose-based medium called Methocult^TM^ GF M3434 (Stem Cell Technology) and cultured at 100 HSCs/ml/well of a 6-well plate. The empty spaces between the wells was filled with 6 ml sterile H2O_dd_. The colonies were analyzed 12 days later.

### Fluorescence labeling of tumor cells or stromal cells

Cells (10^6^) were tagged with either Tag-it violet or CFSE at a dilution of 1:1000 in D-PBS and the cells were added to 1 ml of this solution, incubated for 20 min at 37° in the dark. This was followed by washing with 10 ml D-PBS, centrifugation and resuspending the cells in the appropriate medium. CFSE was used to label the tumor cells in the transwell assay. Tag-it violet was used to label the stromal cells in the HSPC-stromal cell coculture experiments.

### Antibodies and reagents for flow cytometry

These are listed in Method Table I, Method Table II.

**Method Table I:** Antibodies used for flow cytometry

Antigen	Label	Product number	Company	Dilution
CD106 (VCAM)	PerCP-Cy5.5	105715	Biolegend	1:800
CD114	Alexa Fluor 700	FAB60391N-100UG	R&D Systems	1:100
CD117 (c-kit)	PerCP-Cy5.5	105802	Biolegend	1:400
CD11b	Alexa Fluor 700	101222	Biolegend	1:1600
	Biotin	101204	Biolegend	1:400
CD127	PE-Cy7	135014	Biolegend	1:200
CD135	Brilliant Violet 421	135313	Biolegend	1:50
CD144 (VE-Cadherin)	PE-Cy7	138015	Biolegend	1:400
CD146 (MCAM)	APC	134712	Biolegend	1:100
CD150	PE-Cy7	115914	Biolegend	1:800
CD29/Integrin β1	FITC	102206	Biolegend	1:100
CD31	Pacific Blue	102422	Biolegend	1:100
	PE	102508	Biolegend	1:100
	BV510	563089	BD Biosciences	1:100
CD321 (JAM-A)	PE	107803	Biolegend	1:50
CD34	PE	152204	Biolegend	1:200
CD3ε	PE	100308	Biolegend	1:400
CD4	PerCP-Cy5.5	100540	Biolegend	1:400
CD44	APC-Cy7	103028	Biolegend	1:100
Gr1	Biotin	108404	Biolegend	1:400
CD45R/B220	Biotin	103204	Biolegend	1:200
	FITC	103206	Biolegend	1:200
CD45	Brilliant Violet 650	103151	Biolegend	1:400
	APC-Cy7	103116	Biolegend	1:400
	Pacific Blue	103126	Biolegend	1:400
CD48	APC	103411	Biolegend	1:400
CD5	Biotin	100604	Biolegend	1:200
CD54 (ICAM-1)	FITC	116106	Biolegend	1:800
CD8a	Alexa Fluor 700	100730	Biolegend	1:400
CD8a	Alexa Fluor 647	100724	Biolegend	1:800
ESAM	APC	136207	Biolegend	1:50
F4/80	PE	123110	Biolegend	1:400
JAM-C	Biotin	Bs-11086R-Biotin	BIOSS	1:50
LepR	Biotin	BAF497	R&D Systems	1:50
Ly6C	Alexa Fluor 647	128010	Biolegend	1:400
Ly6G	PE-Cy7	127618	Biolegend	1:100
Sca-1	FITC	122506	Biolegend	1:800
	PE-Cy7	122514	Biolegend	1:200
Ter119	Biotin	116203	Biolegend	1:100
	Brilliant Violet 650	116235	Biolegend	1:200
	Pacific Blue	116232	Biolegend	1:200

**Method Table II:** Reagents used for flow cytometry

Reagent	Label	Product number	Company	Dilution
CFSE Cell Division Tracker		423801	Biolegend	1:1000 in DPBS
DAPI		6335.1	Carl Roth	1:1000 in DPBS
Streptavidin	Pacific Orange PerCP-Cy5.5	S32365 405214	Invitrogen Biolegend	1:400 in FaBu 1:400 in FaBu
Tag-it Violet		425101	Biolegend	1:1000 in DPBS
Zombie Aqua Fixable Viability Dye		423102	Biolegend	1:200 in FaBu
Zombie NIR Fixable Viability Dye		423105	Biolegend	1:200 in FaBu
Zombie Red Fixable Viability Dye		423109	Biolegend	1:400 in FaBu
Zombie Violet Fixable Viability Dye		423114	Biolegend	1:400 in FaBu

FaBu: FACS buffer (D-PBS + 1% FCS + penicillin/streptomycin)

### Immune histology

Bones were fixed in 4% PFA for 24 hours, washed, decalcified in 20% EDTA, changed thrice weekly for 2 weeks at 4°C. This was followed by dehydration in 30% sucrose for 48 hours at 4°C and for 24 hours put in a 1:1 solution of 30%sucrose/D-PBS and Tissue-Tek. The bones were transferred to Tissue-Tek and frozen at 20°C and bone sections made using the Kawamoto method [66]. Sections were then blocked using 5% BSA and 0.05% Tween-20 in PBS followed by staining with 100 μl/section of primary antibody from rat directed against CD45 (1:50, clone 30-F11, 550539; BD Pharmingen) in 2.5% BSA/DPBS for one hour, and a FITC-labeled secondary antibody goat anti-rat (Cy2, 1:500, 112-225-003, Dianova), DAPI staining of the nuclei was performed in parallel to the secondary antibody and this step lasted 1 hour in the dark, followed by washing, drying and finally mounting with Mowiol. Sections were evaluated by a scanning laser microscope (Eclipse Ti, Nicon).

### Western blotting

Cells were lysed in a protein lysis buffer (containing 20 mM Tris-HCl, 150 mM NaCl, 10 % Glycerin, 0.5 % Triton X-100, 2 mM EDTA, 10 mM NaF, 1 mM PMSF (Thermo Fisher), 1 mM Na_3_VO_4_, dissolved in dH_2_O), and protein concentration was determined using the BCA-kit (23235, Thermo Scientific) per manufacturers protocol. This was followed by SDS-PAGE using gels containing 8% acrylamide for fibronectin and 10% for β1 integrin. Using the semi-dry method, the proteins were transferred to a membrane before incubation with the following primary antibodies: fibronectin, rabbit, AB2033, Millipore; integrin β1, rat, MB1.2, MAB1997; both from Millipore; GAPDH, rabbit, G9545, Sigma-Aldrich; and either of the secondary antibodies: Goat anti-rabbit, HRP, 111-035-045; or goat anti-rat, HRP, 112-036-071; both from Dianova. Application of ECL Western Blotting-substrate was followed by detection of the chemiluminescence signal with Fusion FX7 (Vilber).

### qPCR

Bone marrow cells were flushed from the long bones of one leg (tibia and femur), the bones were then cut with scissors. A total of 1 ml PEQGold Trifast (Avantor) was used and incubated for 24 hours on a swinging plate at 500 rpm. Following DNA isolation, a two-step PCR was performed. The first included Alu-primers 5’-GGCGGATCACTTGAGGTC, 3’-CGGGTTCAAGCGATTCTCC and the second the following primers: 5’-CATGGTGAAACCCCGTCTCTA, 3’-GCCTCAGCCTCCCGAGTAG, with the following probe: ATTAGCCGGGCGTGGTGGCG [67]. SensiFAST Probe No-ROX-Kit (Bioline, Meridian Bioscience) was used. The results were compared to a standard of MDA-MB-231-B/luc+ cells (each included 10^7^ bone marrow cells with 10/10^2^/10^3^/10^4^ cancer cells). Results were corrected to total murine bone marrow quantified by evaluating murine β-actin with a standard curve of 10^6^/5 × 10^6^/10^7^ bone marrow cells and detected using: 5’-CTAAGGCCAACCGTGAAAAG, 3’-ACCAGAGGCATACAGGGACA, and probe GCAGCCAT. Results are presented as MDA/10^6^ bone marrow cells.

### Cytokine array

In order to determine the concentration of various cytokines in the conditioned media of the various stromal cells, a bead-based Immunoassay LEGENDplex^TM^ (Biolegend) was used according to the manufacturer's protocol. Two panels were used: mouse HSC panel and mouse cytokine release syndrome panel. The following cytokines were tested: IL-34, IL-5, TPO, IL-6, GM-CSF, IL-15, TGF-β1, IL-3, LIF, SCF, EPO, CXCL12, IFN-γ, IL-10, CCL4, CXCL9, IL-4, CCL3 und CCL2.

### Statistical analysis

Statistical analysis of the data was performed using GraphPad Prism software (V10, GraphPad Software Inc.). Outliers were excluded based on a test according to Grubbs (Link: https://www.graphpad.com/quickcalcs/grubbs1/). The significance level was set at α = 0.05. Outliers identified in this process were excluded in subsequent analyses.

Statistical differences between two groups were determined using a two-tailed student's t-test if they were normally distributed, otherwise a nonparametric test (Mann-Whitney) was performed. All correlations were performed using Pearson's correlations. Differences between two experimental groups as a function of a time course were analyzed using regression analysis. If there was a significant difference in the behavior of the groups over time, a post-hoc test was performed. Results are presented as mean± standard error of the mean (M ±SEM).

## Results

### Characterization of promoter activity in the bone marrow

Global deletion of fibronectin or β1 integrin in mice results in early embryonic lethality [24,25]. Therefore, since we aim to elucidate the role of fibronectin and β1 integrin within the bone marrow niche, we chose to use the cre/loxP system. By identifying promoters active in some bone marrow cells, and using these to express cre-recombinase in mice that carry two copies of floxed fibronectin (FN^fl/fl^) or β1 integrin (β1^fl/fl^), the two molecules can be deleted in a variety of bone marrow subpopulations. Since β1 integrin is critical for cell survival, its deletion is expected to lead to loss of some populations. This impairs the ability to confirm its depletion, however [26].

**Fig. 1:**
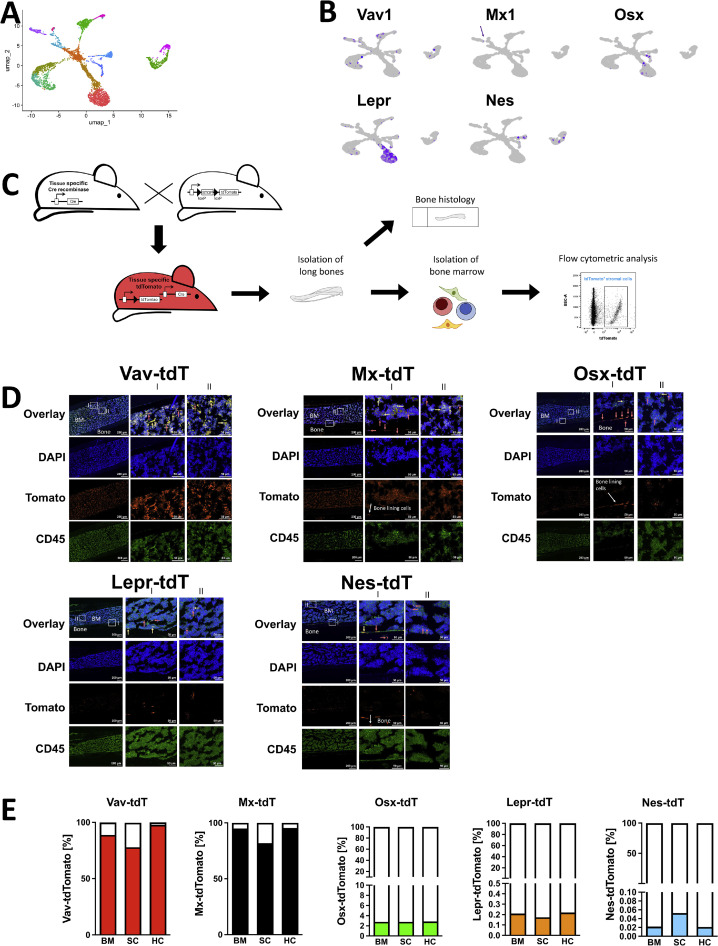
Five promoters attached to cre recombinase are expressed in stromal cells

Several promoters were found to be expressed in various cell types of the bone marrow such as Vav1 and Mx1. In addition, osterix (Osx) is detected in bone lining cells and some CD45^+^ cells, while leptin receptor (Lepr) was used to study the role of stromal cells in hematopoiesis. Finally, nestin (Nes) was detected in few cells of the bone marrow [[27], [28], [29], [30], [31], [32]]. We therefore revisited single-cell RNA sequencing (scRNA-seq) data sets [33], and confirmed the mRNA expression of the five promoters at low levels as shown in the UMAP analysis (Fig. 1a-b). Of note is that Mx1 is an interferon-induced GTP-binding protein. It is normally inactive and hence not expressed (as seen in Fig. 1b). Its promoter can be activated in transgenic mice by injecting polyinosinic:polycytidylic acid (pIpC) replicating RNA stimulation of the promoter to simulate viral infection. In Fig. 1b the promoter was not activated, and hence the low expression, while in Fig. 1d, we had activated the promoter using pIpC. This resulted in cre recombinase production and consequently tdTomato expression in a large number of bone marrow cells [27].

**A.** UMAP visualization of single cell RNA sequencing (scRNA-seq) data showing the relative abundance of the evaluated molecules in the various bone marrow populations. Dataset GSE128423 was analyzed with Seurat package. **B.** Overlay of the expression of the various promoters. Mx1 was not activated and hence little signal is seen (arrow). **C.** Schematic of the detection of cre-expressing cells by use of tdTomato reporter mice. Mice that express cre recombinase under the control of one of the five promoters are mated with animals that carry the tdTomato gene that includes a floxed STOP-codon. Cells that express the promoter and contain the floxed tdTomato gene will remove the STOP-codon, and therefore express the label. These cells can be detected in histology sections or by flow cytometry. The tibia was sectioned, stained for hematopoietic cells (CD45) and examined under the microscope. For flow cytometry, bone marrow is flushed, exposed to a viability marker to exclude dead cells, and stained to detect hematopoietic cells (HC) (CD45^+^) and stromal cells (CD45^-^Ter119^-^). **D.** Images of two areas in longitudinal tibia bone sections for each of the five models to highlight where the promoter is active (the complete longitudinal sections are shown in Supplementary Fig. 1 and larger pictures are reproduced in Supplementary Fig. 2). Both vav and mx are active in most bone marrow cells, while osterix (Osx), leptin receptor (Lepr) and nestin (Nes) are active in fewer cells. The tibia of one leg was isolated from mice expressing cre recombinase under the control of one of the five promoters that also carry the tdTomato gene (this gene includes a floxed STOP-codon). Cells that express the promoter and hence cre-recombinase and in parallel contain the tdTomato gene with the floxed Stop-codon will express tdTomato. The tibia was embedded, sectioned using the Kawamato method, stained for hematopoietic cells (CD45, shown in green), and the nuclei were stained using DAPI (shown in blue). The cells that have an active promoter and hence express tdTomato appear in red, unless they also express CD45, in which case they appear yellow. Red arrows point to tdTomato-expressing cells; i.e., cells in which cre recombinase is expressed. Yellow arrows point to yellow cells, which represent the green CD45^+^ hematopoietic cells that also express cre recombinase and are therefore also red, where green+red gives a yellow color. White arrow points to bone lining cells in Mx and Osx mice and to cells in the cortical bone in Nes mice. **E.** Flow cytometry evaluation confirms expression of tdTomato in total bone marrow (BM), stromal cells (SC), and hematopoietic cells (HC) in all models to various degrees. N=7/7 mice (Vav), 6/6 (Mx), 12/12 (Osx), 8/8 (Lepr), 5/5 (Nes). BM: bone marrow, SC: stromal cells, HC: hematopoietic cells.

Next, we evaluated which bone marrow subpopulations were affected by the different promoters using tdTomato reporter mice [34]. Cells in which the promoter of interest is active will produce tdTomato (tdT) that can be detected on histology sections or by flow cytometry (Fig. 1c). These mice are compared to tdTomato reporter mice lacking cre recombinase expression. Bone sections showed marked expression of tdTomato in the bone marrow in Vav- and Mx-tdT mice, while bone marrow involvement in Osx-, Lepr- and Nes-tdT was less pronounced. Involvement of bone lining cells was seen in Mx- and Osx-tdT. In addition, Nes-tdT was expressed in cells within the cortical bone (red or yellow cells in Fig. 1d and Supplementary Fig. 1 and 2). These findings were confirmed by examining the flushed and stained bone marrow (BM) by flow cytometry. Stromal cells (SC: CD45^-^Ter119^-^) and hematopoietic cells (HC: CD45^+^) could thus be differentiated (Fig. 1e). This analysis confirmed activity of the promoters in stromal cells to various degrees in the five models.

### Efficiency of fibronectin or β1 integrin deletion

**Fig. 2:**
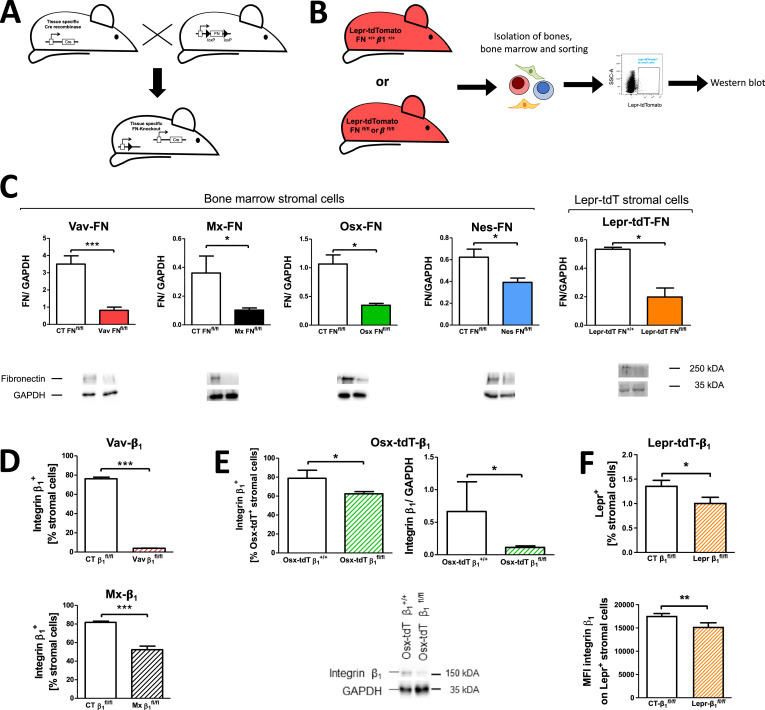
Depletion of fibronectin and β1 integrin in the various transgenic mouse models

The five promoter-cre lines were used to delete either fibronectin (in FN^fl/fl^ mice) or β1 integrin (in β1^fl/fl^ mice). The names of the transgenic lines were abbreviated to promoter-FN or promoter-β1 for the respective conditional knockout mice, e.g. Vav-FN or Vav-β1 for the vav promoter line (Fig. 2a) [[32], [35],^36^].

**A.** Schematic showing the strategy of taking advantage of the promoters attached to cre that are activated in bone marrow cells to delete genes containing loxP sites on both alleles. Shown is the example for fibronectin floxed mice. **B.** Schematic showing the strategy to sort cells that are homozygous for a floxed gene such as fibronectin or β1 integrin, by taking advantage of tdTomato gene expression. If the promoter attached to cre is expressed (in this case the leptin receptor (Lepr) promoter), the fluorophore will be produced allowing sorting and at the same time the floxed gene will be depleted in the same cells. **C. Depletion of fibronectin.** Fibronectin is decreased in the bone marrow stromal cells as detected by western blotting of bone marrow stromal cell lysates (after removal of CD45^+^ cells) in vav-cre, mx-cre, osterix-cre and nestin-cre, while confirmation of depletion in lepr-cre required sorting of the cre-expressing cells as detailed in B. This approach confirmed depletion of fibronectin in the Lepr^+^-sorted cells. N= 9/10 (Vav), 4/6 (Mx), 11/9 (Osx), 5/5 (Nes), 5/4 (Lepr). Differences between the control (CT) group and the transgenic group were evaluated using student's t-tests. *p<0.05, **0<0.01, ***p<0.005. **D-F. Depletion of β1 integrin. D.** β1 integrin is depleted in the bone marrow stromal cells as detected by flow cytometry in vav-cre and mx-cre mice. [N=6/6 (vav), 23/21 (mx)]. **E.** In osterix-β1 mice (Osterix-^cre/+^_tdTomato^fl/+^_β1 integrin^fl/fl^), mice that express osterix-cre as well as the reporter fluorophore tdTomato were generated as shown in Fig. 2B, and cells expressing the promoter were sorted. Flow cytometric (left graph) and western blotting analysis of these cells (right graph and picture of western blot) confirmed depletion of β1 integrin, N=4/6. **F.** Evaluation of loss of β1 integrin in Lepr-stained cells. A decrease in the percentage of Lepr^+^ cells (in relation to stromal cells) was detected, but the low prevalence prevented seeing any changes in relation to the total bone marrow (less than 0.2%). In addition, the mean fluorescence intensity of β1 integrin was diminished on Lepr^+^ cells. N=11/11. Differences between the control (CT) group and each transgenic group were evaluated using student's t-tests. *p<0.05, **p<0.01, ***p<0.005.

Depletion of fibronectin (FN) was confirmed by analyzing cell lysates of isolated total stromal bone marrow cells in Vav-FN, Mx-FN, Osx-FN and Nes-FN (Fig. 2c). In Lepr-FN, the cells with activated promoter were initially sorted based on tdTomato expression. Comparison of fibronectin expression between control mice (Genotype: Lepr-^cre/+^_ tdTomato^fl/+^_ FN^+/+^) and mice homozygous for floxed fibronectin (Lepr-FN: Lepr-^cre/+^_ tdTomato^fl/+^_ FN^fl/fl^; right bar in Fig. 2b-c) confirmed reduced fibronectin in sorted Lepr-FN cells (Fig. 2c).

β1 integrin depletion in Vav-β1 and Mx-β1 models was readily confirmed in the bone marrow stromal cells by flow cytometry (Fig. 2d), while depletion in Osx-β1 required the use of reporter mice homozygous for floxed β1 integrin according to the schematic illustrated in Fig. 2b for Lepr-FN mice (Genotype: Osterix-^cre/+^_tdTomato^fl/+^_β1 integrin^fl/fl^). This approach confirmed a decrease in β1 integrin on tdTomato cells (and hence Osx-expressing cells) by flow cytometry and allowed confirmation of depletion in sorted Osx-β1 cells by western blotting (Fig. 2e). Nestin-mediated depletion of β1 was lethal within 5 weeks after birth. Lastly, depletion of β1 integrin using the Lepr promoter (Lepr-β1) led to a reduction in the number of Lepr-stained cells and diminished the mean fluorescence intensity (MFI) for β1 integrin (Fig. 2f), presumably due to partial loss of β1 integrin-depleted cells.

In summary, depletion of fibronectin was confirmed across all five models, as was depletion of β1 integrin in Vav-, Mx-, and Osx-β1 mice. The Nes-β1 model was excluded from further study due to early lethality, and the Lepr-β1 model exhibited a reduction in Lepr^+^-cells and in β1 integrin surface expression.

### Fibronectin and β1 integrin effects on homing of cancer cells

**Fig. 3:**
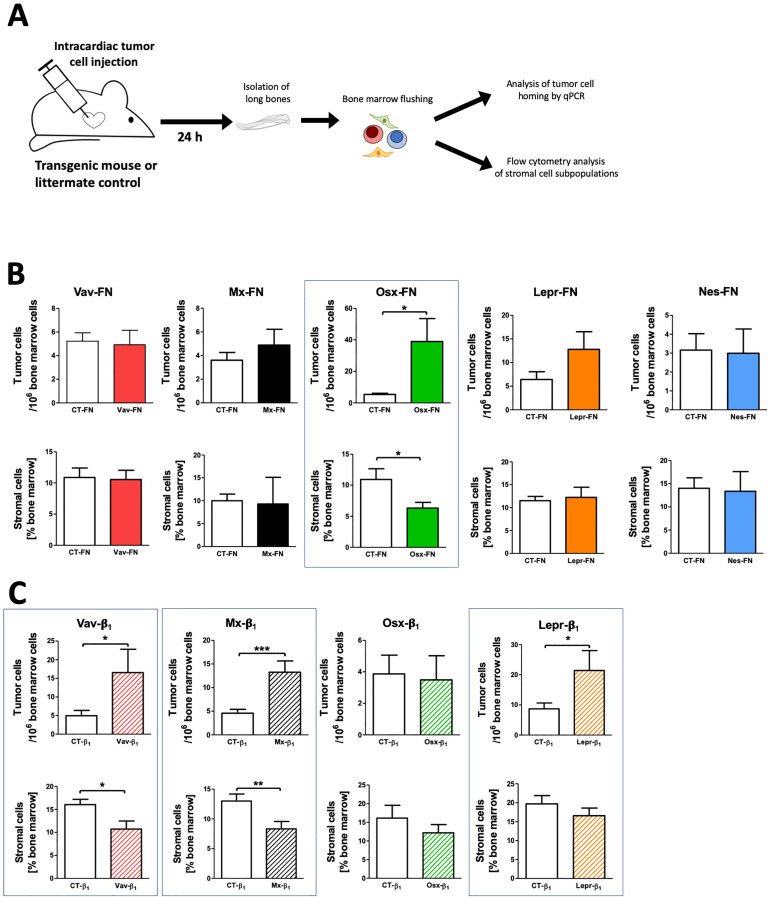
Influence of fibronectin or β1 depletion in various stromal cells on tumor cell homing to the bone marrow

Fibronectin (FN) is a chemotactic factor [4]. Therefore, its production by stromal cells may play a role in modulating tumor cell migration to the bone marrow. To test this, Vav-FN, Mx-FN, Osx-FN, Lepr-FN, and Nes-FN mice were compared to control (CT) littermate animals (FN^fl/fl^). Tumor cells were injected intracardially and the number of tumor cells in the bone marrow was quantified 24 hours later using qPCR (Fig. 3a). Only osterix-mediated depletion of fibronectin (Osx-FN) was associated with increased tumor cell number in the bone marrow (Fig. 3b).

**A.** Schematic of the experimental design. Tumor cells were injected in transgenic mice intracardially. Twenty-four hours later, bone marrow was collected and the number of tumor cells was assessed by qPCR. The characteristics of the stromal cells were examined by flow cytometry and compared to stromal cells isolated from control littermate mice (CT). **B.** Of the five models with depletion of fibronectin, only lowering fibronectin in osterix-expressing mice (Osx-FN) resulted in increased tumor cell homing to the bone marrow (upper row in Fig. 3B). Stromal cells were also decreased in this model, but not in the other four fibronectin models (bottom row in 3B). **C.** Depletion of β1 integrin in three models (Vav-β1, Mx-β1 and Lepr-β1), but not in Osx-β1, resulted in enhanced homing of tumor cells to the bone marrow, A decrease in stromal cells in the Vav-β1 and Mx-β1 models, but not in the Lepr-β1 model was seen. N for FN: 22/21 (Vav), 22/21 (Mx), 22/22 (Osx), 17/19 (Lepr), 19/18 for homing and 18/13 for stromal cells (Nes); N for β1 integrin: 23/12 (Vav), 23/21 (Mx), 22/15 (Osx), 26/16 for homing and 44/43 for stromal cells (Lepr). Differences between the control (CT) group and the transgenic group were assessed using student's t-tests. *p<0.05, **p<0.01, ***p<0.005.

Fibronectin can bind to various integrins, predominantly those containing the β1 subunit. The same genetic approach was used in conditional knockout β1 integrin mice. While diminishing β1 integrin using the osterix promoter did not affect the number of tumor cells detected in the bone marrow, Vav-, Mx-, and Lepr-β1 increased homing (Fig. 3c).

Thus, of the 9 models evaluated, four were associated with increased homing, namely: Osx-FN, Vav-β1, Mx-β1 and Lepr-β1.

### Identification of a population inversely correlated with tumor cell homing to the bone marrow

An inverse correlation between tumor cell numbers and a subpopulation of stromal cells in the bone marrow in mice has been reported [13]. In line with this, two stromal cell subpopulations were diminished in patients afflicted with breast or prostate cancer in whom tumor cells were found in the bone marrow [13]. We therefore evaluated whether there is a relationship between increased homing and diminished stromal cells in our transgenic mouse models.

In three of the four models exhibiting enhanced tumor cell homing to the bone marrow (Osx-FN, Vav-β1 and Mx-β1), stromal cell percentages were reduced (Fig. 3b and c, lower rows). This decrease was not related to the injection or presence of tumor cells in the bone marrow, because it was also detected in mice that did not receive tumor cells (Supplementary Figs. 3 and 4). Since we aim to correlate stromal cells with tumor cells, stromal cell subpopulations shown in the context of tumor cell injection were quantified in the bone marrow of mice that received tumor cell injections. Even though proliferation or apoptosis were not affected (Supplementary Fig. 5), the decrease in stromal cells is probably attributable to effects mediated by the depletion of the gene(s) in the respective cell types.

**Fig. 4:**
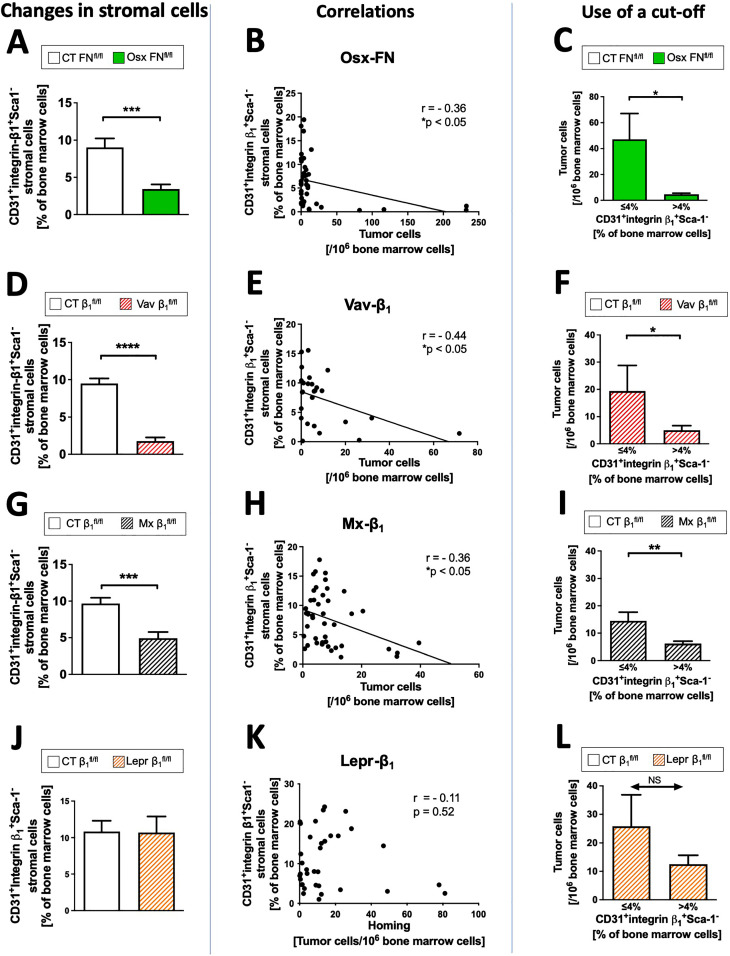
Finding the stromal cell population associated with increased homing

Across these three genotypes, the subpopulations expressing CD31^+^, β1 integrin^+^, or not expressing Sca1 (Sca1^-^), as well as the combination of all three characteristics were reduced relative to total bone marrow in mice exposed to cancer cells (Supplementary Figs. 3 and 4, Fig. 4a, d, g). Interestingly, the three subpopulations or the combined one represented a major portion of stromal cells and were close in size raising the possibility that they all represent the same cells.

**A, D, G. J. Changes in stromal cells.** Evaluation of the subpopulations that changed in the three models with increased homing and decreased stromal cells confirms that only three subpopulations were diminished, namely, Sca-1^-^, CD31^+^ and integrin β1^+^ as shown in the supplementary figure 3 and 4. Shown here is the combination of the three relevant markers. The graphs are shown in A for Osx-FN, D for Vav-β1, and G for Mx-β1. Cells isolated from the bone marrow at the time of killing 24 hours after tumor cell injection were stained with a viability marker, CD45 and Ter119 to exclude hematopoietic cells, as well as various stromal markers. N= 22/22 (Osx-FN), 23/12 (Vav-β1), 23/21 (Mx-β1). Differences between the control (CT) group and the transgenic group were evaluated using student's t-tests. ***p<0.005, ****p<0.001. J. Lepr-β1 mice do not have a decrease in the stromal subpopulation CD31^+^ integrin β1^+^ Sca-1^-^ seen in the three other models with increased homing in A, D and G. N=22/13. **B, E, H, K. Correlations.** Tumor cell homing negatively correlated with the newly identified subpopulation characterized as CD31^+^β1 integrin^+^Sca-1^-^ in the three models with increased homing (Osx-FN, Vav-β1, Mx-β1), but not in Lepr-β1 mice shown in K. The genotype is shown above each graph: Osx-FN (N=40 pairs), Vav-β1 (N=26 pairs), Mx-β1 (N=43 pairs), Lepr-β1 (N=35 pairs). Pearson's correlation was evaluated and linear regression applied to show the regression line for statistically significant correlations. *p<0.05. **C, F, I, L. Use of a cut-off.** The number of tumor cells detected in the bone marrow is lower whenever the subpopulation CD31^+^ integrin β1^+^ Sca-1^-^ exceeds 4% in relation to total bone marrow cells in the three models (Osx-FN, Vav-β1, Mx-β1), but not in Lepr-β1 (L). N= 16/24 (Osx-FN), 7/19 (Vav-β1), 15/28 (Mx-β1), 7/28 (Lepr-β1). Differences between two groups were evaluated using student's t-tests. *p<0.05, **p<0.01.

No changes in percentages of stromal cells in naïve mice were detected in Lepr-β1 mice, despite enhanced homing (Supplementary Fig. 6A, Fig. 4j), or in the other five models in which homing was not affected (Supplementary Fig. 6B-C and Supplementary Fig. 7A-C). Changes in the various subpopulations 24 hours after tumor cell injection are summarized in Table 1. In line with this, The stromal subpopulation correlated negatively with tumor cell homing to the bone marrow in the three models, but not in Lepr-β1 mice (Fig. 4b, e, h, k). Therefore, mice with lower percentages of this stromal subpopulation harbor more tumor cells in the bone marrow than mice with higher stromal cell percentages. Various cutoffs were evaluated (Supplementary Table 1), and a cutoff between 2 and 5% of the stromal subpopulation resulted in significant separation in mean tumor cell numbers in the bone marrow in the three models. Interestingly, all mice of the genotype Vav-β1 had less than 4% of the stromal cell subpopulation and all controls had a percentage of the subpopulation that is higher than 4%. Using this cutoff of 4% for the population of CD31^+^ integrin β1^+^ Sca1^-^ cells, shows that a low percentage is associated with more tumor cells in the bone marrow, except in Lepr-β1 (Fig. 4c, f, i, l).

**Table 1:** Summary of homing of cancer cells to the bone marrow and changes in the various **stromal cell subpopulations** in the bone marrow in relation to total bone marrow for the transgenic mouse models evaluated 24 h after tumor cell injection. All arrows denote statistically significant changes with p<0.05 or lower. Cells with an increase are colored grey and those with a decrease are highlighted in green.

**Fig. 5:**
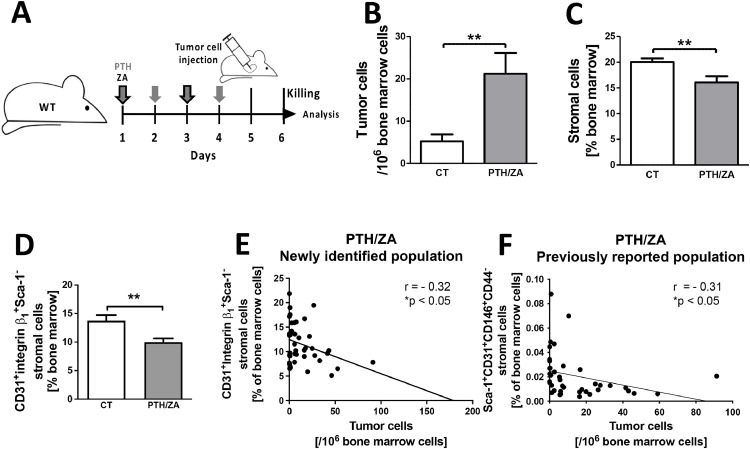
Pharmacological manipulation of the niche

We had found that a pharmacologic intervention to modulate the osteoblasts and osteoclasts using parathyroid hormone (PTH) and zoledronic acid (ZA), followed by intracardiac tumor injection on day 5, and subsequent quantification of tumor cell numbers 24 hours later on day 6 (as illustrated in Fig. 5a), resulted in a decrease in stromal cells in the bone marrow with a concurrent increase in tumor cell count (Fig. 5b-c) [13]. We re-evaluated the bone marrow stromal cells in this model to include the new markers examined in the transgenic mice. Alongside increased homing, the changes in the stromal subpopulations showed similarities to those observed in the three transgenic mouse models (Supplementary Fig. 8 and Fig. 5d). Both the newly identified CD31^+^ integrin β1^+^ Sca1^-^ population and the previously identified population of the non-hematopoietic CD31^+^Sca-1^+^CD146^+^CD44^-^ bone marrow cells correlated with tumor cell homing in the same mice in the PTH/ZA model, while in the other four models the previously identified subpopulation did not show any relationship to tumor cell numbers (Fig. 5e-f and Supplementary Fig. 9) [13]. Notably, the new population represents roughly 5-10% of bone marrow cells, which is larger than the population reported in the pharmacologic model, and which constituted only 0.2% (Fig. 5E-F, compare y-axis for both graphs). Hence, the new population is more accessible.

**A.** Schematic of the experimental design: Wildtype mice were injected subcutaneously with 400 ng/g/day parathyroid hormone (PTH) to support osteoblast proliferation and survival on days 1-4. In parallel, mice received 100 ng/g zoledronic acid (ZA) injected intraperitoneally on days 1 and 3 to prevent osteoclastic bone resorption and release of growth factors stored in the bone. On day 5 tumor cells were injected intracardially, and 24 hours later, on day 6, the bone marrow was evaluated for the number of tumor cells detected in relation to total nucleated bone marrow cells by qPCR. In addition, bone marrow cells were stained using a viability stain, CD45 and Ter119 to exclude hematopoietic cells, as well as stromal cell markers similar to those used for the transgenic mouse models. **B.** Treatment with PTH/ZA leads to more tumor cells detected in the bone marrow. **C.** Increased homing shown in B is associated with decreased total stromal cells in the bone marrow after PTH/ZA administration. **D.** The change in the stromal subpopulation that is diminished in the transgenic mice models is also present after PTH/ZA treatment 24 hours after tumor cell injection. N=20/22 for B-D. Percentages were compared using student's t-test. **p<0.01. **E-F. Correlations.** E shows the correlation with the newly identified stromal cell subpopulation and F shows the correlation with the previously published subpopulation, both are listed on the Y-axis (N=42 pairs). Pearson's correlation was evaluated and linear regression performed to show the regression line when the correlation was statistically significant. *p<0.05.

### Changes in CD31 expression underlie enhanced homing in the transgenic mouse models

We had already established causality between an increase in bone marrow stromal cells and suppressed homing [13], but stromal cells were diminished in only three out of the four transgenic models showing enhanced homing. Even though in the fourth model, Lepr-β1, a reduction in Lepr-expressing cells in relation to stromal cells was found (Fig. 2f), this was not the case in relation to total bone marrow cells (Supplementary Fig. 6A and Table 1). This raises the possibility that another mechanism might be at play.

**Fig. 6:**
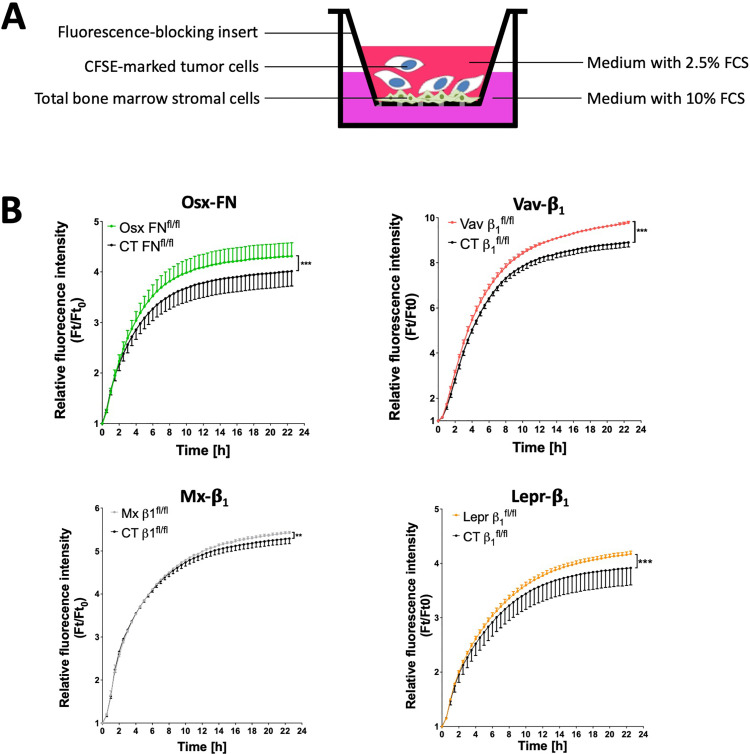
*In vitro* studies to evaluate the mechanisms of increased tumor cell migration to the bone marrow

**Fig. 7:**
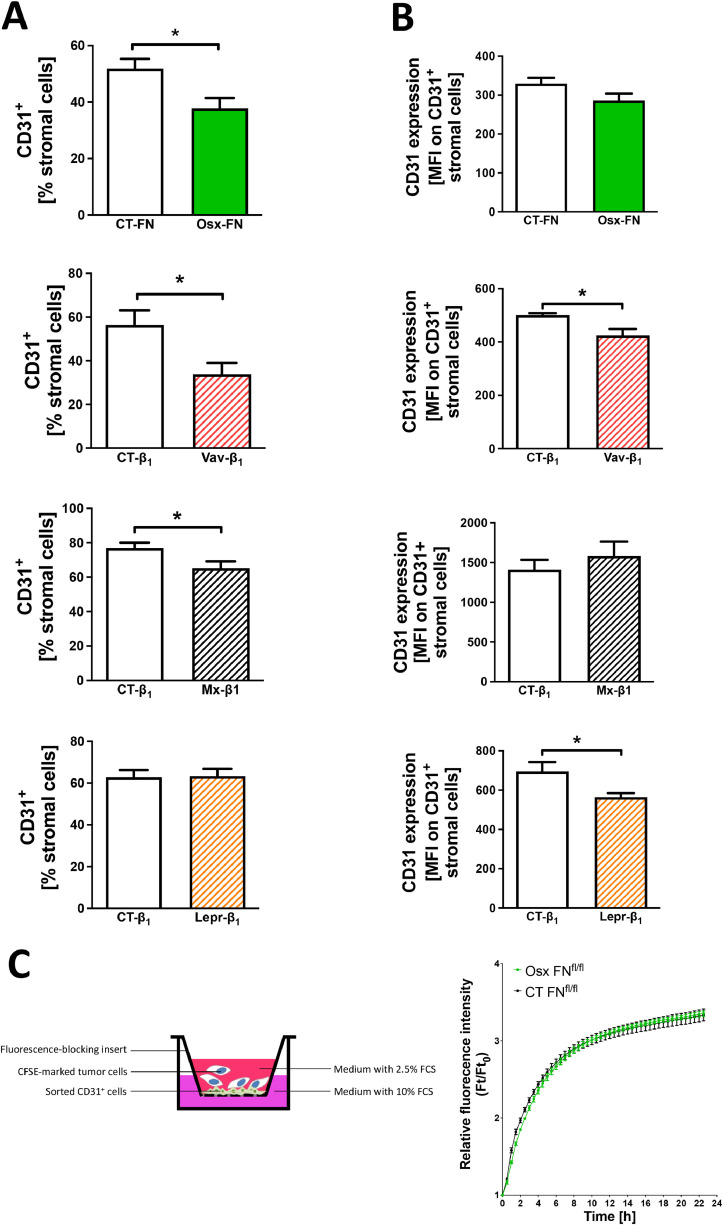
CD31 expression in the bone marrow stromal cells of the models associated with increased *in vitro* migration

We therefore first assessed whether there is a change in the number of CD31^+^-stained vessels in the four models with increased homing, but could not detect any difference (Supplementary Fig. 10). We next examined tumor cell migration through a stromal cell layer isolated from the bone marrow of the four models: Osx-FN, Vav-β1, Mx-β1 and Lepr-β1, and found that migration was enhanced in all (Fig. 6a-b). One possible explanation could be a change that facilitates tumor cell migration through the vascular barrier. In search of changes in molecules affecting transmigration of cells through the vascular barrier, six molecules were evaluated (Supplementary Table 2). A reduction in either the percentage of CD31-expressing cells and/or the expression of CD31 on stromal cells was the only common change across the four models (Fig. 7a-b and Supplementary Table 2). Increased transmigration was not due to intrinsic changes in the CD31^+^ cells, since culturing sorted CD31^+^ cells led to an almost perfect overlap in transmigration (Fig. 7c).

**A.** Schematic of the experimental design. Total bone marrow stromal cells were cultured for 24 hours in inserts to form a confluent layer and placed in an appropriately sized well of a plate. Labeled tumor cells were added in the insert in medium containing 2.5% FCS. Migration of the tumor cells through the stromal cell layer on the insert toward the bottom well containing 10% FCS was evaluated over the following 24 hours. **B.** Transmigration of tumor cells through the stromal cell layer was increased in all four models with enhanced tumor cell homing to the bone marrow. This suggests that migration is facilitated in the absence of fibronectin or β1 in the stromal cells. N=10/10 (Osx-FN), N=3/3 (Vav-β1), N=3/3 replicates (Mx-β1), N=3/5 replicates (Lepr-β1) across three separate experiments. Migration was evaluated using non-linear regression analysis. *p<0.05, ***p<0.005.

**A.** Changes in the percentage of cells expressing CD31 in relation to total stromal cells. **B.** Alterations in the mean fluorescence intensity of CD31 on CD31^+^ stromal cells. Bone marrow stromal cells were flushed and stained with a viability marker to exclude dead cells, CD45 and Ter119 to exclude hematopoietic cells, and CD31. N= 11/11 (Osx-FN), 12/13 (Vav-β1), 11/15 (Mx-β1), 43/44 in A and 4/5 in B (Lepr-β1). Values were compared using student's t-test. *p<0.05. **C.** Schematic showing that the cells cultured in the insert are sorted CD31^+^ cells. 700 000 cells were added to each insert, cultured for 24 hours before adding the tumor cells in the insert and evaluating transmigration to the bottom well containing 10% FCS. The graph shows an almost complete overlap between the two curves. N=4/2 in two experiments for Osx-FN. Migration was evaluated using non-linear regression analysis.

In summary, increased homing results from the relative decrease in CD31^+^ numbers and/or expression in a bone marrow subpopulation.

### Establishment of metastasis is independent of the number of tumor cells in the bone marrow

**Fig. 8:**
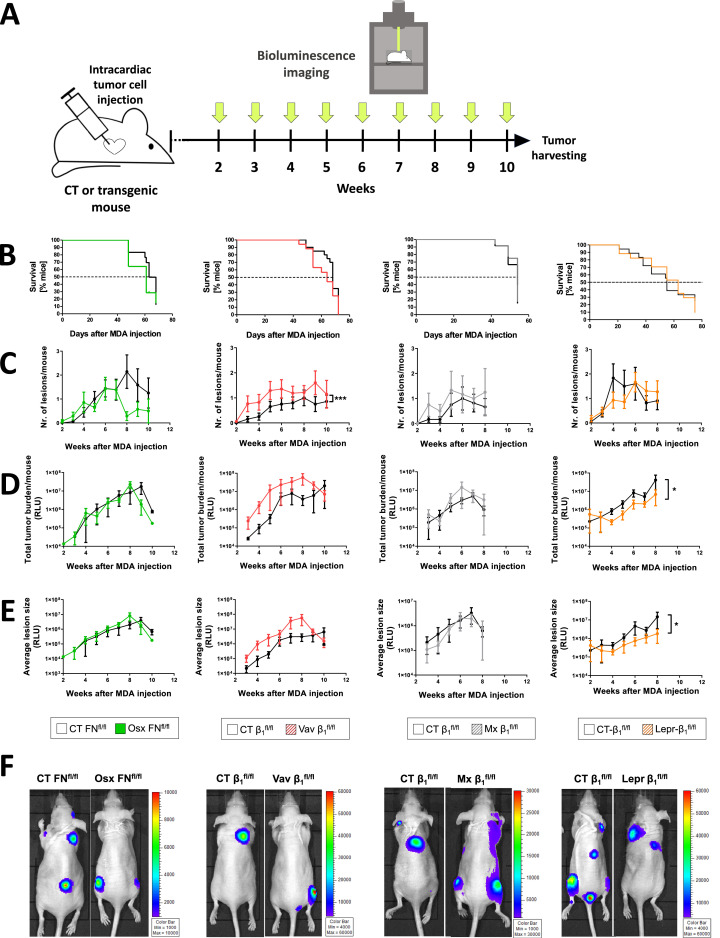
Relationship between increased homing and tumor progression

An increase in the number of tumor cells in the bone marrow increases the likelihood of cancer lesions in patients [37,38]. We therefore evaluated cancer growth following intracardiac injection in the four models exhibiting increased homing (Fig. 8a). Since the cell line used, MDA-MB-231-B/luc^+^, only homes to the bone marrow, metastases consisted of bone lesions only [39]. Survival, the number of lesions, the tumor burden (as evidenced by increased bioluminescence signal (relative light units: RLU)), or the average size of lesions (total burden in RLU divided by the number of lesions) were all evaluated. This was made possible by the expression of luciferase in the tumor cell line used [[6], [39], [40]]. Localization of all bioluminescence signals to bones was confirmed at the time of killing.

**A.** Schematic showing the experimental design to evaluate cancer progression. Tumor cells were injected intracardially in the mouse models exhibiting increased homing. Starting on week 2, bioluminescence imaging was performed weekly to determine tumor burden. **B.** Survival was not compromised in any model associated with increased homing. **C.** The number of lesions over time did not differ either, except in Vav-β1 mice, where it was higher compared to littermate controls. **D.** The total tumor burden was similar in all groups, except for Lepr-β1, which showed a decrease instead of the expected increase compared to CT. **E.** The average size per lesion was only changed in the Lepr-β1 model, where it was smaller than in controls. **F.** Examples of bioluminescence imaging. After intracardiac injection of tumor cells, growth was evaluated weekly by means of bioluminescence imaging. N=18/14 (Osx-FN), 20/17 (Vav-β1), 12/12 (Mx-β1), 10/11 (Lepr-β1). Raw data measurements from bioluminescence imaging are presented as relative light units (RLU), which is proportional to the number of photons detected. Kaplan-Meier analysis was used to examine survival. Curves were compared using non-linear regression analysis. *p<0.05, ***p<0.005.

Survival was not diminished in any of the models (Fig. 8b). Vav-β1 was the only model that resulted in the expected development of more metastatic lesions (Fig. 8c), although the tumor burden (RLU/mouse) remained unchanged. Unexpectedly, in Lepr-β1 mice, survival was unaffected despite a decrease (instead of the expected increase) in both tumor burden and average lesion size (Fig. 8d-f). Limiting the analysis to the lesions in the hind limbs only confirmed an increase in tumor burden and average lesion size in Osx-FN and Vav-β1, but a decrease in these two parameters in Lepr-β1 (Supplementary Fig. 11).

In summary, it is not the number of tumor cells in the bone marrow, but the microenvironment that dictates tumor progression. Furthermore, despite the small size of the Lepr^+^ population, it seems to contribute to tumor growth.

### Transgenic mouse models associated with changes in hematopoiesis

In search of a potential overlap between the metastatic and hematopoietic niche, we evaluated the following populations in hematopoiesis: early hematopoietic stem cells (HSCs), multipotent progenitors (MPPs), as well as the combination of HSCs and MPPs called hematopoietic stem and progenitor cells (HSPCs), the common myeloid and common lymphoid progenitors (CMP and CLP), the granulocyte-monocyte progenitors (GMPs) and megakaryocyte-erythroid progenitors (MEP) (Supplementary Fig. 12) [41].

The three models which showed increased homing and decreased stromal cells, namely Osx-FN, Vav-β1, and Mx-β1, demonstrated enhancement of various stages of myelopoiesis, while Lepr-β1 did not (Fig.s 9a-c, Supplementary Fig. 13 and Table 2).

**Fig. 9:**
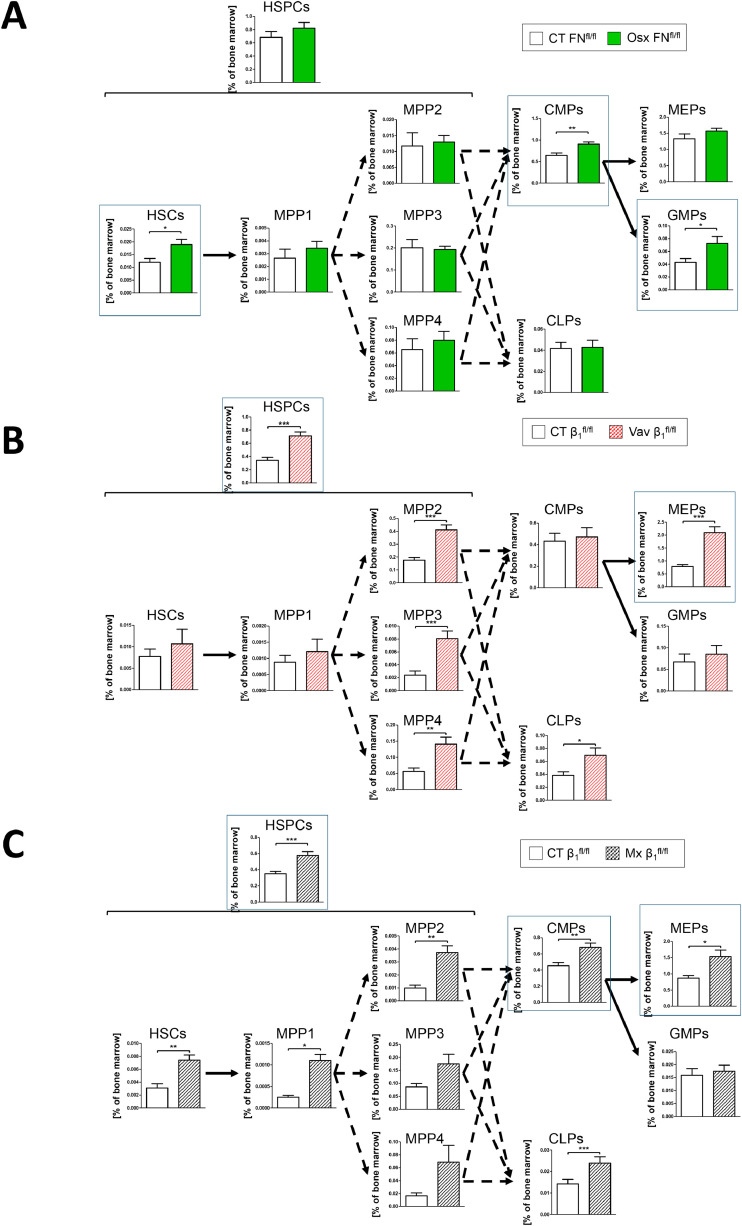
Hematopoiesis in the models with increased homing and decreased stromal cells

**A-C.** Changes in myelopoiesis are seen. In Osx-FN mice, evaluation of hematopoiesis reveals an increase in HSCs, CMPs and GMPs **(A)**, while Vav-β1 is associated with increased HSPCs and MEPs **(B)**, and Mx-β1 shows increases in HSCs, HSPCs, CMPs and MEPs **(C)**. Early hematopoietic stem cells (HSCs) and multipotent progenitors (MPPs) were evaluated in the bone marrow separately from evaluation of hematopoietic stem and progenitor cells (HSPCs), common myeloid progenitors (CMPs), common lymphoid progenitors (CLPs), megakaryocyte-erythroid progenitors (MEPs), and granulocyte-monocyte progenitors (GMPs). N=9/14 for HSCs and MPPs, and 20/23 for HSPC and the rest (Osx-FN), N=10/13 for HSCs and MPPs, and 20/21 for HSPC and the rest (Vav-β1), N=6/10 for HSCs and MPPs, and 16/22 for HSPC and the rest (Mx-β1). Data were compared using student's t-tests. *p<0.05, **p<0.01, ***p<0.005.

**Table 2:** Summary of changes in hematopoiesis in all transgenic mouse models. The following cells are reported: hematopoietic stem cells (HSC) and multipotent progenitors (MPP), their combination in hematopoietic stem and progenitor cells (HSPC), and their differentiation to common lymphoid progenitors (CLP), common myeloid progenitors (CMP), megakaryocyte-erythroid progenitors (MEP), and granulocyte-monocyte progenitors (GMP). Arrows denote statistically significant changes at the time of analysis at the ages of 5-6 weeks relative to control littermate animals not carrying the promoter. To facilitate visualization, cells with an increase in transgenic mice compared to littermate controls are colored green. The orange cells in this table are to be cross-referenced with corresponding cells in Table 3.

**Fig. 10:**
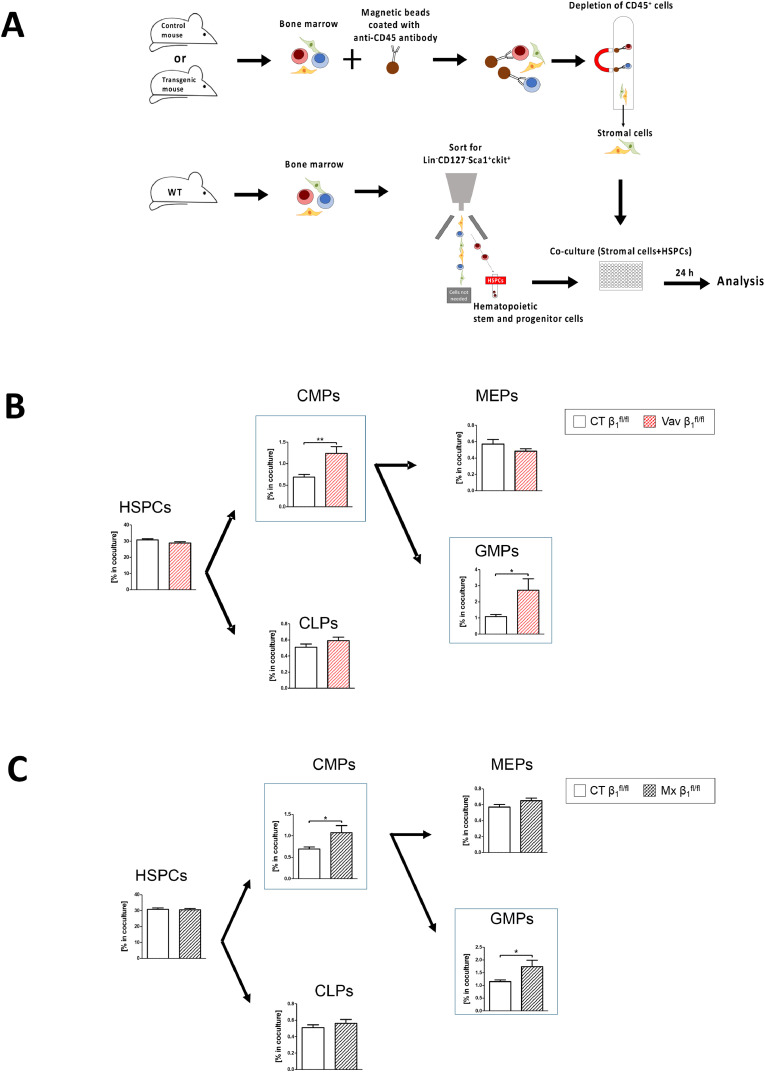
Impact of transgenic stromal cells on hematopoiesis

Since all promoters used were active both in stromal cells and hematopoietic CD45^+^ cells (as quantified in Fig. 1e and Supplementary Fig. 2B), we performed *in vitro* experiments using stromal cells from the transgenic mouse models cocultured with sorted wildtype HSPCs (Fig. 10a). Osx-FN stromal cells did not elicit any changes (Table 3). This may be attributed to the low percentage of osx-cells in the flushed bone marrow, or potentially to the relatively short duration of the culture (24 hours) compared to the *in vivo* scenario. In contrast, Vav-β1 and Mx-β1 stromal cells promoted myelopoiesis, aligning with the changes seen *in vivo* in Osx-FN mice (compare Fig. 10b-c with Fig. 9a, data summarized in Table 3).

**A.** Schematic representation of the experiment used for characterization of the contribution of stromal cells from transgenic mice to hematopoiesis *in vitro*. Stromal cells were isolated from the bone marrow of transgenic mice and their littermate controls (5 × 10^5^ cells), labeled and co-cultured for 24 hours with hematopoietic stem and progenitor cells (HSPCs: Lineage^-^ckit^+^Sca-1^+^) sorted from wildtype mice (5 × 10^4^) in medium containing 2.5% FCS (Gating strategy for sorting is shown in Supplementary Fig. 12C). When using fibronectin conditional knockout cells, the FCS used was depleted of fibronectin. Hematopoiesis was evaluated 24 hours later after exclusion of the pre-labeled stromal cells. **B.** Stromal cells derived from Vav-β1 mice enhanced CMP and GMP percentages compared to stromal cells from littermate controls. N=17/20. **C.** Similarly, stromal cells from Mx-β1 also showed an increase in CMP and GMP compared to their littermate controls. N=16/18. Hematopoietic stem and progenitor cells (HSPC), common lymphoid progenitors (CLP), common myeloid progenitors (CMP), megakaryocytic-erythroid progenitors (MEP), and granulocytic monocytic progenitors (GMP) were examined. Data were compared using student's t-tests. *p<0.05, **p<0.01.

**Table 3:** Summary of findings of *in vitro* differentiatio**n** of sorted hematopoietic stem and progenitor cells (HSPC). HSPCs, common myeloid progenitors (CMP), megakaryocyte-erythroid progenitors (MEP), granulocytic-monocytic progenitors (GMP), and common lymphoid progenitors (CLP) are presented. Arrows denote statistically significant changes at the time of analysis of cells isolated from mice aged 5-6 weeks compared to cells from control littermate animals not carrying the promoter. Stromal cells obtained from the various transgenic animals were cocultured with HSPCs isolated from wildtype animals for 24 hours (Left). HSPCs from transgenic animals were cocultured with stromal cells isolated from bone marrow of wildtype animals. In both instances, the cocultures lasted for 24 hours and were evaluated by flow cytometry. To facilitate visualization, cells with an increase are colored green and those with a decrease are blue. The orange cells should be compared with the orange cells in Table 2. cKO: conditional knockout for fibronectin (FN) or β1 integrin, depending on the promoter used. The transgenic models are listed in the left column.

Increased homing and myelopoiesis in the same three transgenic models suggest an overlap in the stromal cells constituting the myelopoietic niche and those making up the pre-metastatic niche.

Further evaluation of hematopoiesis revealed several alterations summarized in Table 2, Table 3 and Fig. 10. Notably, depletion of β1 integrin in hematopoietic cells using the vav promoter led to enhanced retention of hematopoietic stem cells (HSCs) in the bone marrow (Table 2 and Supplementary Fig. 14).

Cytokines were analysed in the conditioned media of stromal cells isolated from the bone marrow of the four models (Osx-FN, Vav-β1, Mx-β1 and Lepr-β1). No consistent changes could be detected across the four models with enhanced homing or the three models exhibiting changes in myelopoiesis (Osx-FN, Vav-β1, Mx-β1). This suggests that either these cytokines were not involved or that different mechanisms are at work in the different models (Supplementary Table 3).

In conclusion, there is partial overlap between the pre-metastatic and the hematopoietic stem cell niche.

## Discussion

**Fig. 11:**
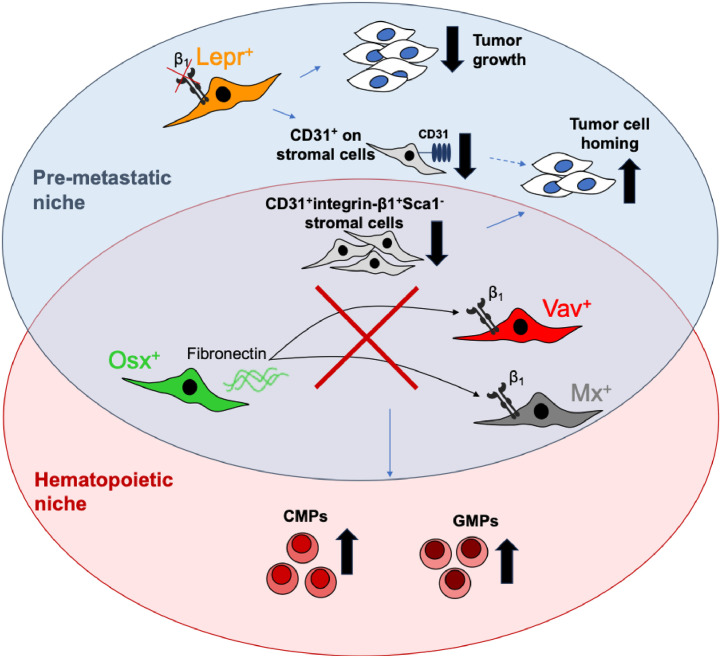
Partial overlap between the pre-metastatic and hematopoietic niche.

The findings of this study indicate that the pre-metastatic niche and the hematopoietic niche in the bone marrow partially overlap. The overlapping part consists of stromal cells that express the transcription factor osterix (sp7) and produce fibronectin, as well as cells capable of activating the promoters of vav and mx1 and that display β1 integrin on their surface (Fig. 11). In these three models both homing and myelopoiesis were increased. The fourth model, Lepr-β1 was similarly associated with increased homing, but failed to affect hematopoiesis. Instead, suppressed tumor growth was found. The reason for the increase in the number of cancer cells that manage to get into the bone marrow was a decrease in CD31^+^ cells or expression in a stromal subpopulation. These results therefore shed light on the composition of the two niches and establish a role for fibronectin and β1 integrin in both.

**(In the overlapping part):** Depletion of fibronectin in osterix-expressing cells in the bone marrow cells or β1 integrin in vav- or mx- expressing cells leads to a decrease in the percentage of a stromal population that contains CD31^+^ cells resulting in enhanced transmigration through cell barriers and increased homing of tumor cells to the bone marrow. **(In the premetastatic niche only):** The depletion of β1 integrin on leptin receptor (Lepr) cells diminishes CD31^+^ expression on the cells. This ultimately enhances transmigration and homing. This depletion is also associated with suppressed tumor growth. **(In the hematopoietic niche):** In the three models: osterix, vav and mx, stromal cells not only affect homing, but additionally modulate myelopoiesis. Thus, fibronectin and β1 integrin in stromal cells regulate the differentiation of common myeloid progenitors (CMP) and granulocyte-monocyte progenitors (GMP).

Much has been learned from the study of the premetastatic niche, where cancer lesions produce factors and vesicles that in turn prepare organs to become recipients for circulating tumor cells [42]. The organs to which they can home and develop metastases differ depending on the cancer cell type and its characteristics. This contrasts with the model presented in this work. The cancer cells used were selected to get into the bone marrow within 24 hours after intracardiac injection and form bone metastatic lesions [39]. While our model does not allow to recapitulate the complete metastatic cascade starting with a primary tumor, it allows for a focused evaluation of the role of specific molecules or stromal cell subpopulations in the bone marrow on cancer development and progression in the bone.

Various cell types have been implicated as components of bone marrow niches, the role of two of these have been extensively characterized: osteolineage cells and vascular cells [43]*.* Since osterix is expressed in osteoblast progenitors it can be considered part of the so called endosteal/osteoblastic niche. Our data in Osx-FN mice support the importance of the osteoblastic niche in homing and hematopoiesis [[44], [45], [46]]. In particular, fibronectin production by these cells regulates niche function. Vav on the other hand is expressed in endothelial cells [47], which is in line with the relevance of CD31 in characterizing the bone marrow pre-metastatic niche seen in our model and the involvement of endothelial cells in supporting hematopoiesis [48,49].

It is surprising that loss of fibronectin, a chemoattractant, enhances tumor cell migration to the bone marrow. Fibronectin, however, is also a pro-survival factor [50]. Therefore, one potential explanation is that its loss leads to depletion of a subpopulation that would otherwise impede migration of tumor cells into the bone marrow. Indeed, in all murine models with enhanced homing (Fig. 3, Fig. 5) either the percentage of cells expressing CD31 (also known as PECAM1) or the mean expression levels on these cells was diminished (Fig. 7 and Supplementary Fig. 8). This change points out to a reduction in the integrity of the vascular wall, facilitating translocation of tumor cells across the endothelial barrier [51], and is in line with the reported proximity of breast cancer tumor cells in the bone marrow to blood vessels [52]. Endothelial cells that do not express Sca-1 represent a differentiated cell population [53] and might be part of the blood vessel barrier. Similarly, deletion of β1 integrin leads to loss of some populations [26], and jeopardizes the integrity of the vessel walls [54]. This is also consistent with work by others showing that necroptosis of endothelial cells by activation of death receptor 6 (DR6) enhanced metastasis formation [55]. Attributing increased migration through stromal cells to the decrease in a subpopulation that expresses CD31 or the amount of CD31-receptors on this subpopulation identifies a unifying mechanism for enhanced tumor migration not only in the mice, but also in patients with breast and prostate cancer, in whom the presence of tumor cells in the bone marrow was associated with diminished CD31 expression [13].

Detecting tumor cells in the bone marrow in patients with breast or prostate cancer at the time of diagnosis is associated with cancer progression and a poor prognosis [[56], [57], [58]]. It was therefore surprising that despite enhanced homing in four models, none developed a larger total tumor burden (Fig. 8), even though larger lesions developed in Osx-FN and Vav-β1 in the hind limbs (Supplementary Fig. 11). Instead, tumor growth was diminished in the Lepr-β1 model (despite the presence of more tumor cells). This supports the conclusion that Lepr^+^ cells represent a component of the pre-metastatic niche that modulates the development of bone macrometastases. It is possible, that Lepr^+^ cells represent part of recruited fibroblasts to the tumor and that β1 integrin in these cells is needed to support growth (Fig. 8). Thus, not only is β1 expression in tumor cells supportive of cancer progression, but it may also modulate the behavior of stromal cells recruited by the tumor [59]. Notably, Lepr^+^ cells were shown to represent part of the hematopoietic stem cell niche and to modulate hematopoiesis [60]. Based on our findings, however, neither fibronectin nor β1 integrin expression in these cells had any effect on hematopoiesis. Furthermore, it was reported that SCF (Stem cell factor) and CXCL12 production by Lepr^+^ cells mediate persistence of hematopoietic stem cells in the bone marrow raising the possibility that these molecules are similarly involved in enhancing tumor cell homing in Lepr-β1 mice [48,61]. However, neither SCF nor CXCL12 were affected (Supplementary Table 3) excluding a contribution for these molecules in increased homing in Lepr-β1 mice. Even though a metastatic signature has been reported in cancer cells that develop metastases [62], our data support the conclusion, that the microenvironment modulates tumor cell behavior. This underscores the need for a thorough characterization of the metastatic niche in the bone marrow before we can explore novel approaches to suppress cancer progression in bone.

The changes in hematopoiesis mainly consisted of enhancement of myelopoiesis and were seen in three out of four mouse models with increased homing. Fibronectin produced by osterix cells modulates myelopoiesis *in vivo*, but stromal cells from these Osx-FN mice failed to show any changes in myelopoiesis *in vitro*. This could be due to a variety of reasons such as the low percentage of affected cells (Fig. 1e). Another possible explanation is that fibronectin originating from bone lining cells (Fig. 1d) is critical for myelopoiesis, but flushed bone marrow does not contain these cells. Because this was not seen in Vav-FN or Mx-FN mice, even though mx also affects bone lining cells, it is possible that the isoforms produced differ based on the promoter the cells express. Indeed, in previous work, we found that loss of an isoform (EDA-fibronectin) increased the myeloid progenitor population GMP, as seen here [45].

The ability of the stromal cells from Vav-β1 and Mx-β1 mice to stimulate myelopoietic differentiation of hematopoietic stem and progenitor cells (HSPCs) isolated from wildtype mice *in vitro* establishes a role for these stromal cells in modulating myelopoiesis (Table 2). For this, fibronectin may act on β1 integrin located on stromal cells. We had also seen an increase in myelopoiesis by deleting cdc42, a Rho GTPase, in osx-cre expressing mice [46]. This therefore adds yet another molecule to a hypothetical cascade of fibronectin possibly acting on β1 integrin on stromal cells to then activate cdc42 and ultimately leads to downregulation of myelopoiesis. No changes in hematopoiesis/myelopoiesis were seen in Lepr-β1, even though these cells are part of the pre-metastatic niche. One possibility is that β1 integrin in these cells does not affect myelopoiesis, either because of the low expression of Lepr in the bone marrow or because these cells are not relevant for myelopoiesis.

## Conclusions

In addition to defining a stromal subpopulation that mediates increased homing of cancer cells to the bone marrow, combined changes in homing and hematopoiesis were seen in Osx-FN, Vav-β1 and Mx-β1. In contrast, Lepr-β1 showed an increase in homing and suppression of tumor growth but no changes in hematopoiesis. Thus, some overlap exists between the hematopoietic and pre-metastatic niche, but this overlap is not complete. Furthermore, the overlap is not limited to the stem cell niche and might also be related to the niche of differentiating hematopoietic cells, particularly those along the myeloid pathway. Our study thus highlights the importance of stromal cells in tumor establishment and hematopoiesis, and sheds some light on the role of two key molecules fibronectin and β1 integrin in these processes. These findings, in particular regarding the dependence of cancer progression on the microenvironment, highlight the need for a better understanding of the bone marrow niches in order to identify new strategies for targeting metastatic lesions.

## Funding Sources

German Research Council (DFG: NA400/9; NA400/10-401246035; Max-Planck Society (M.KF.A.BIOC0001).

## CRediT authorship contribution statement

**Franziska Wirth:** Writing – review & editing, Visualization, Methodology, Investigation. **Caren Zoeller:** Writing – review & editing, Visualization, Methodology, Investigation. **Alexander Lubosch:** Writing – review & editing, Visualization, Validation, Investigation. **Jutta Schroeder-Braunstein:** Writing – review & editing, Methodology. **Guido Wabnitz:** Writing – review & editing, Visualization, Methodology, Investigation. **Inaam A. Nakchbandi:** Writing – review & editing, Writing – original draft, Visualization, Supervision, Resources, Project administration, Methodology, Funding acquisition, Conceptualization.

## Declaration of competing interest

The authors declare that they have no known competing financial interests or personal relationships that could have appeared to influence the work reported in this paper.

## Appendix Supplementary materials

Image, application 1
