# Supplementary material for: Insights into the metastatic bone marrow niche gained from fibronectin and β1 integrin transgenic mice
Source: Neoplasia. 2024 Oct 15;58:101058. doi: 10.1016/j.neo.2024.101058 (PMC11530925; doi:10.1016/j.neo.2024.101058)
Supplement: Supplementary file 1 [file mmc1.pdf]

INSIGHTS INTO THE METASTATIC AND HEMATOPOIETIC BONE MARROW NICHES GAINED FROM FIBRONECTIN AND β1 INTEGRIN TRANSGENIC MICE

[*§]Franziska Wirth, [*§]Caren Zoeller, [*]Alexander Lubosch, [*]Jutta Schroeder-Braunstein, [*]Guido Wabnitz, [*†&]Inaam A. Nakchbandi

[*]Institute of Immunology, Heidelberg University, 69120 Heidelberg, Germany,
[†]Max-Planck Institute for Biochemistry, 82152 Martinsried, Germany,
[&]Max-Planck Institute for Medical Research, 69120 Heidelberg, Germany.

[§]The two authors contributed equally to this work

Running Title: Fibronectin and β1 integrin in metastatic and hematopoietic niche

Supplementary Figure 1

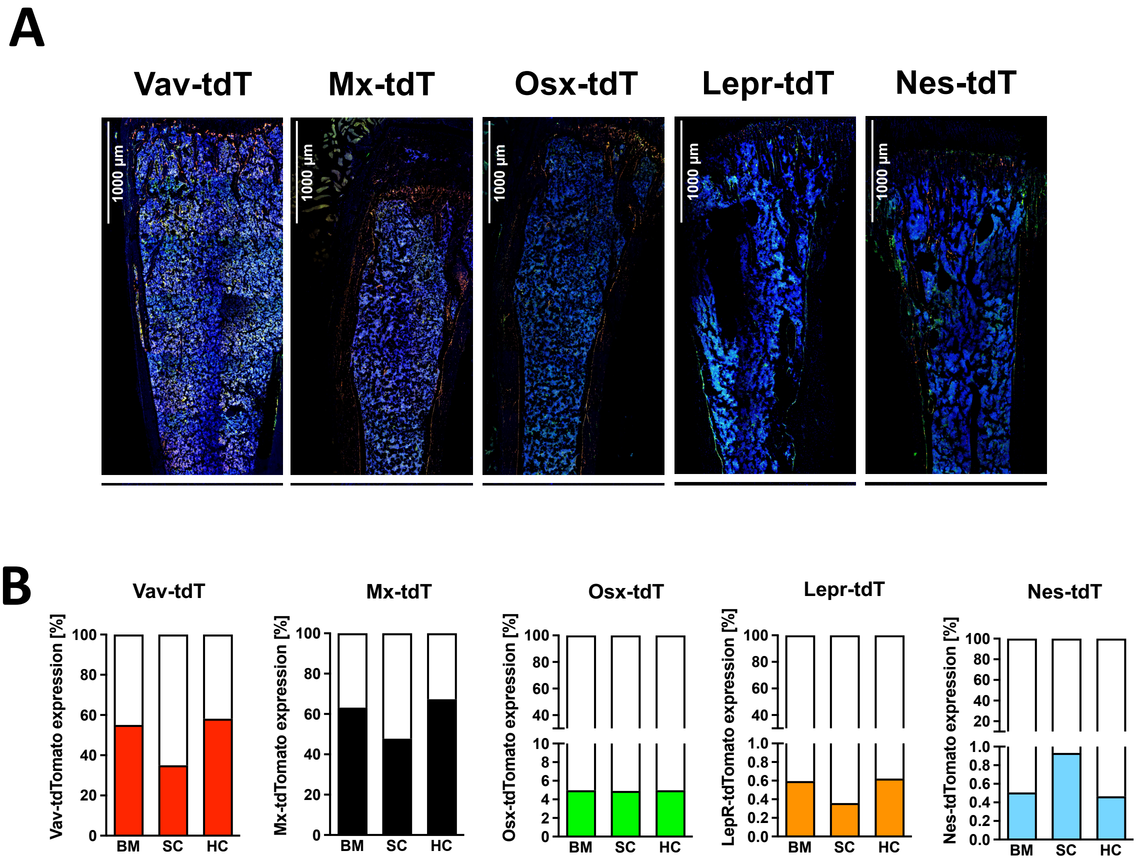

Supplementary Figure 1.

Histology evaluation of bone sections using the five promoters.

A. Longitudinal tibia sections of mice generated as shown in the schematic in Figure 1C illustrate the differences in promoter expression. Red-colored cells represent stromal cells that express the promoter and therefore can be used for deletion of fibronectin or β1 integrin, green represents stained hematopoietic $CD45^+$ cells and yellow represents hematopoietic cells that also express the promoter (green and red). Blue denotes DAPI-stained nuclei. The tibia was sectioned using the Kawamato method, stained and analyzed using laser scanning microscopy. Higher magnification images are shown in Figure 1D and Supplementary Figure 2.

B. Quantitative analysis of sections: in the first column of each graph (bone marrow: BM), the combination of tdTomato- (red-) expressing stromal cells (SC) and yellow $CD45^+$ hematopoietic cells (HC: $CD45^+$) are shown in relation to total nucleated bone marrow cells. Yellow cells are green $CD45^+$ cells that express the red tdTomato fluorophore. Some differences in the percentages are detected compared to flow cytometry results (shown in Figure 1E). N=3-4 mice for either the littermate controls or the cre-expressing mice of each genotype. The area evaluated is similar to that evaluated in bone histomorphometry. It starts 150 μm below the growth plate and extends distally for 1.5 mm as described (Bentmann, Kawelke et al. 2010, Huck, Sens et al. 2020). BM: bone marrow (red + yellow) to total nucleated cells, SC: stromal cells represented by the red cells in relation to the combination of cells that are red + cells that are not green (blue nuclei), HC: hematopoietic cells (These are the yellow cells - green and red simultaneously- in relation to all $CD45^+$ cells (green and yellow).

Supplementary Figure 2

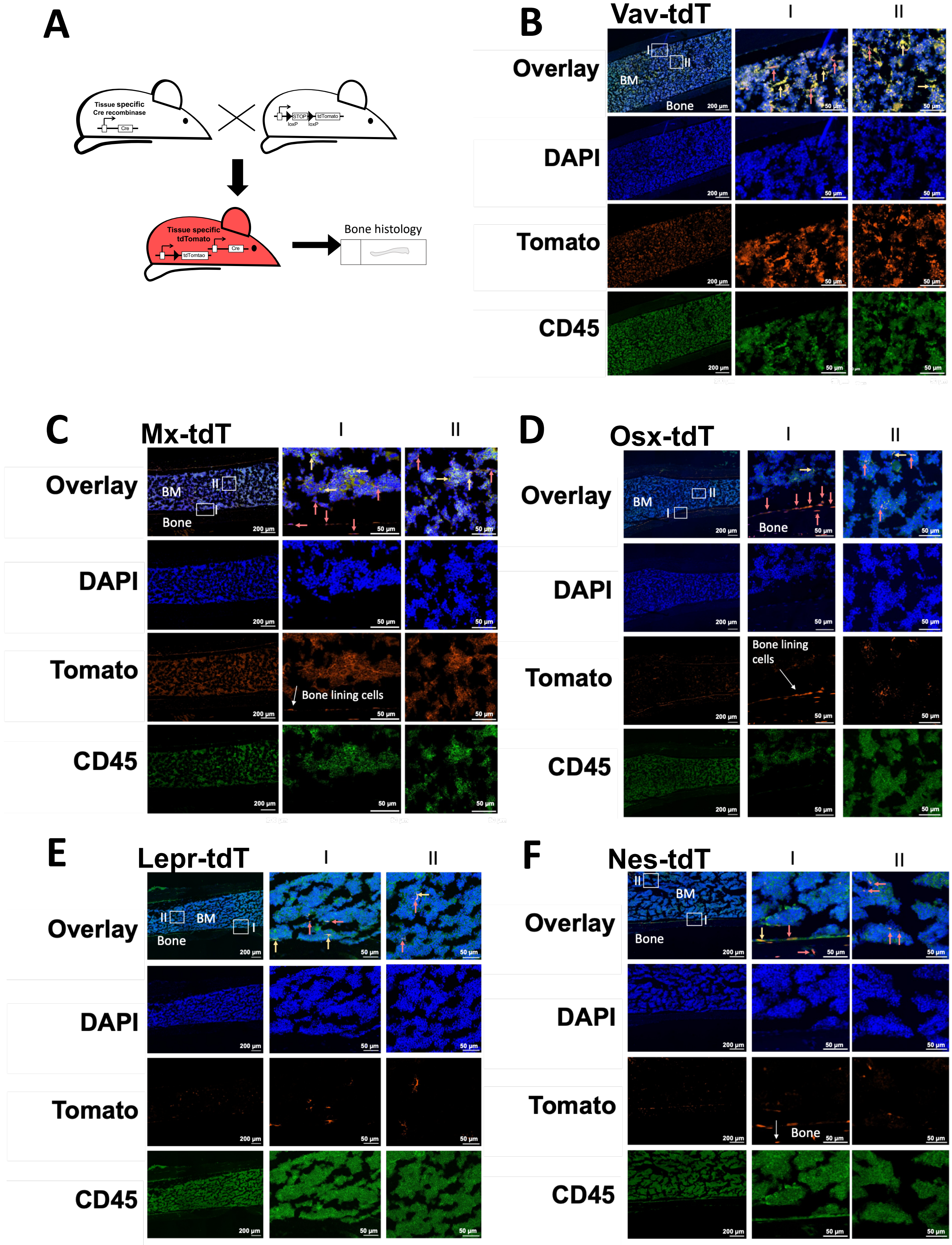

Supplementary Figure 2.
Expression of the promoters in bone sections
A. Schematic of the detection of cre-expressing cells by use of tdTomato reporter mice. Mice that express cre recombinase under the control of one of the five promoters are mated with animals that carry the tdTomato gene that includes a floxed STOP-codon. Cells that express the promoter will produce cre recombinase which will remove the STOP-codon if the cell also contains the floxed tdTomato gene. Removal of the STOP-codon will lead to expression of the label. These cells can be detected in histology sections (shown in red). B. High magnification images of two areas in longitudinal tibia bone sections for each of the five models shown in Figure 1D are enlarged here. Both vav and mx are active in most bone marrow cells, while osterix (Osx), leptin receptor (Lepr) and nestin (Nes) are active in fewer cells. The tibia of one leg was isolated from mice expressing cre recombinase under the control of one of the five promoters that also carry the tdTomato gene (this gene includes a floxed STOP-codon). The tibia was embedded, sectioned using the Kawamato method, stained for hematopoietic cells (CD45, shown in green), and the nuclei were stained using DAPI (shown in blue). The cells that have an active promoter and hence express tdTomato appear in red, unless they also express CD45, in which case they appear yellow. Red arrows point to tdTomato-expressing cells; i.e., cells in which cre recombinase is expressed. Yellow arrows point to yellow cells, which represent the green $CD45^{+}$ hematopoietic cells that also express cre recombinase and are therefore also red, where green+red gives a yellow color. White arrows point to bone lining cells in Mx and Osx mice and to cells in the cortical bone in Nes mice. BM: bone marrow.

Supplementary Figure 3
Tumor cells present

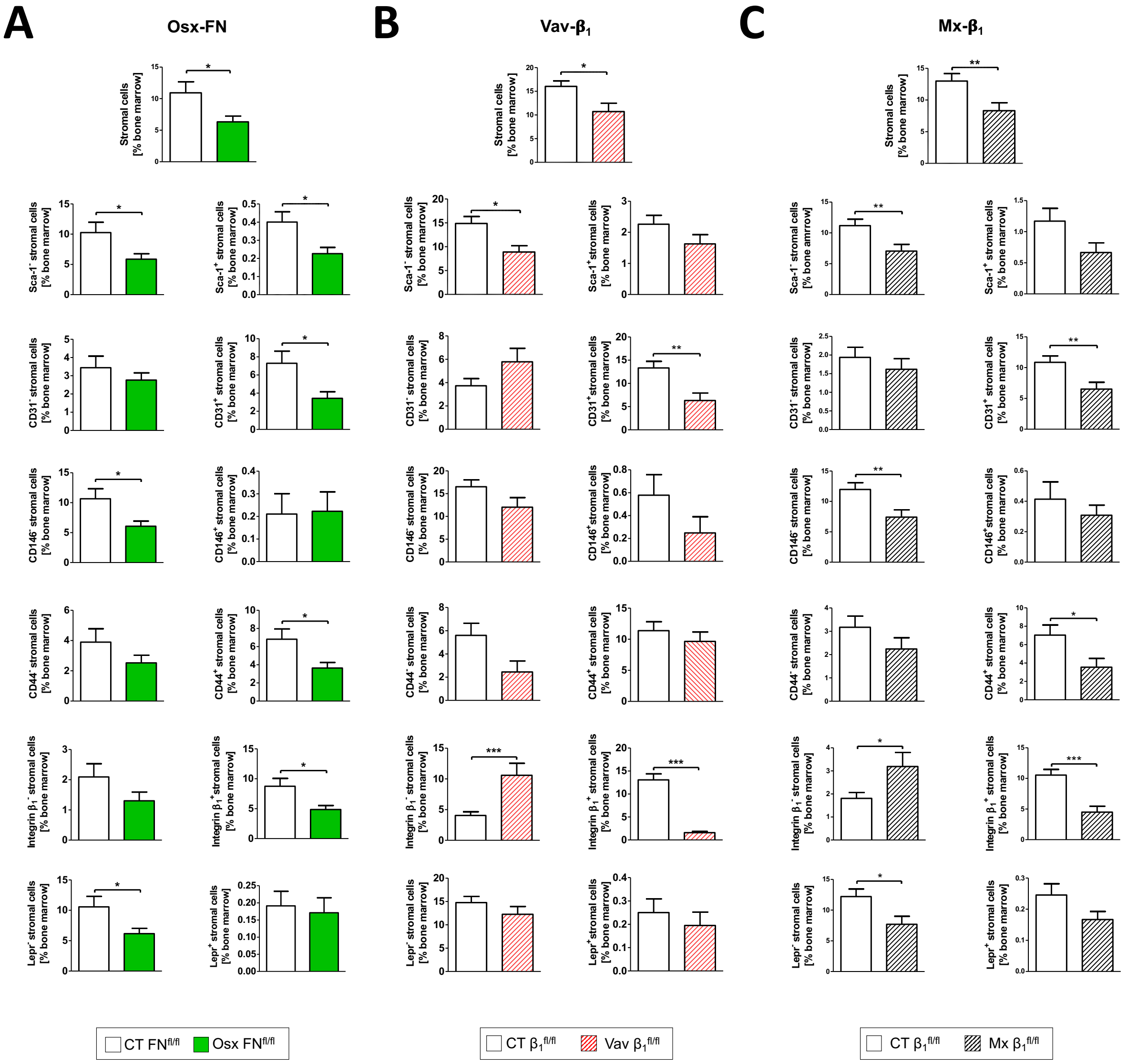

Supplementary Figure 3.
Changes in markers on stromal cells in relation to the total bone marrow <u>in mice after tumor cell injection</u> in the three models associated with increased homing and decreased stromal cells (Osx-FN, Vav-β1 and Mx-β1)

*A. Changes in markers on stromal cells for Osx-FN, B. Changes in markers on stromal cells for Vav-β1. C. Changes in markers on stromal cells for Mx-β1. Cells isolated from the bone marrow were stained with a viability marker, CD45 and Ter119 to exclude hematopoietic cells, as well as various stromal markers. N= 22/22 (Osx-FN), 23/12 (Vav-β1), 23/21 (Mx-β1). Percentages were compared using student's t-tests. *p<0.05, **p<0.01, ***p<0.005.*

Supplementary Figure 4
No tumor cells

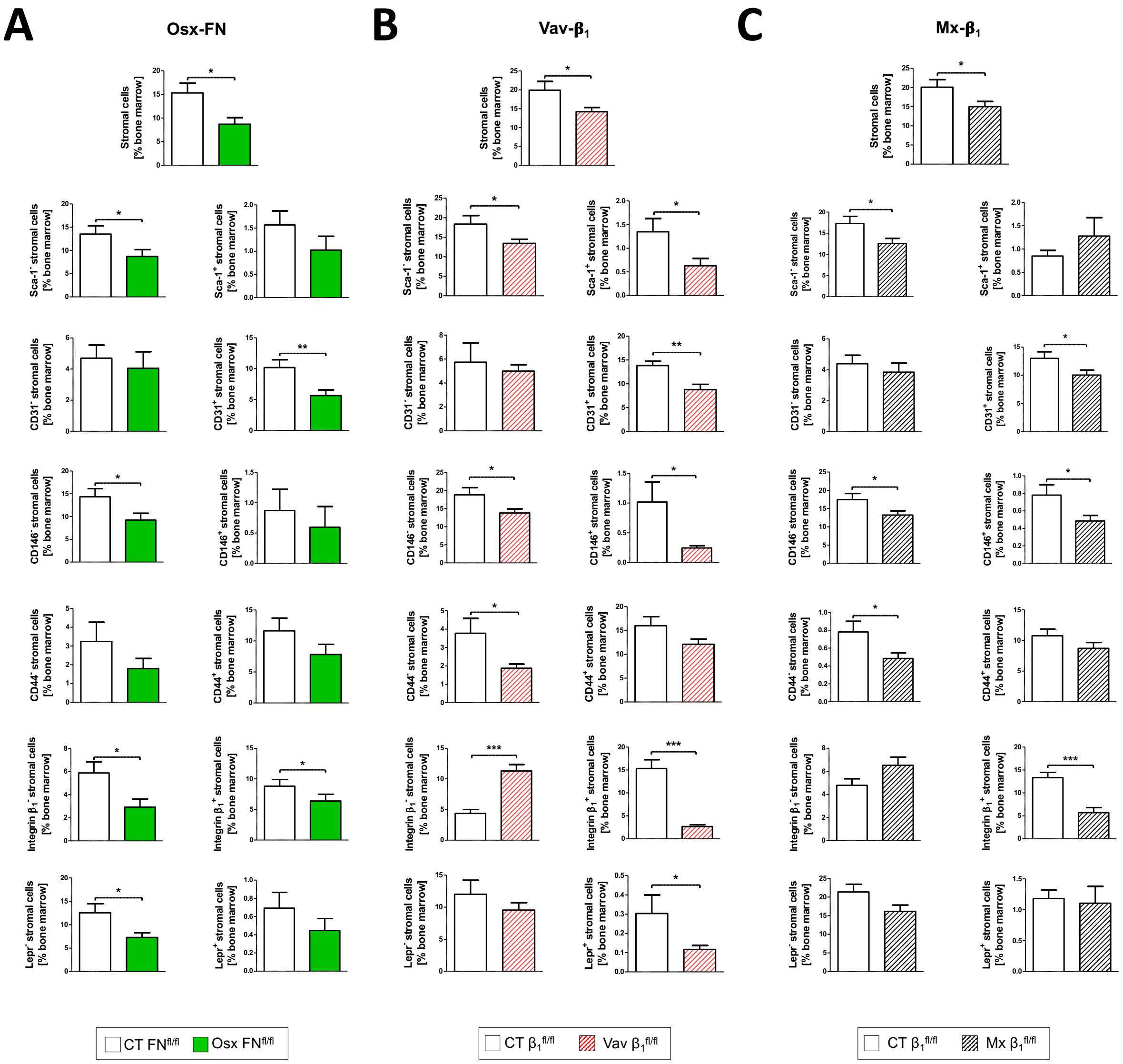

Supplementary Figure 4.

Changes in markers on stromal cells in relation to the total bone marrow in the three models associated with increased homing and decreased stromal cells (Osx-FN, Vav-β1 and Mx-β1) <u>in mice that were not exposed to cancer cells</u>

*A. Changes in markers on stromal cells for Osx-FN, B. Changes in markers on stromal cells for Vav-β1. C. Changes in markers on stromal cells for Mx-β1. Cells isolated from the bone marrow were stained with a viability marker, CD45 and Ter119 to exclude hematopoietic cells, as well as various stromal markers. N= 21/17 (Osx-FN), 19/21 (Vav-β1), 19/22 (Mx-β1). Percentages were compared using student's t-tests. *p<0.05, **p<0.01, ***p<0.005.*

Supplementary Figure 5
Proliferation and apoptosis

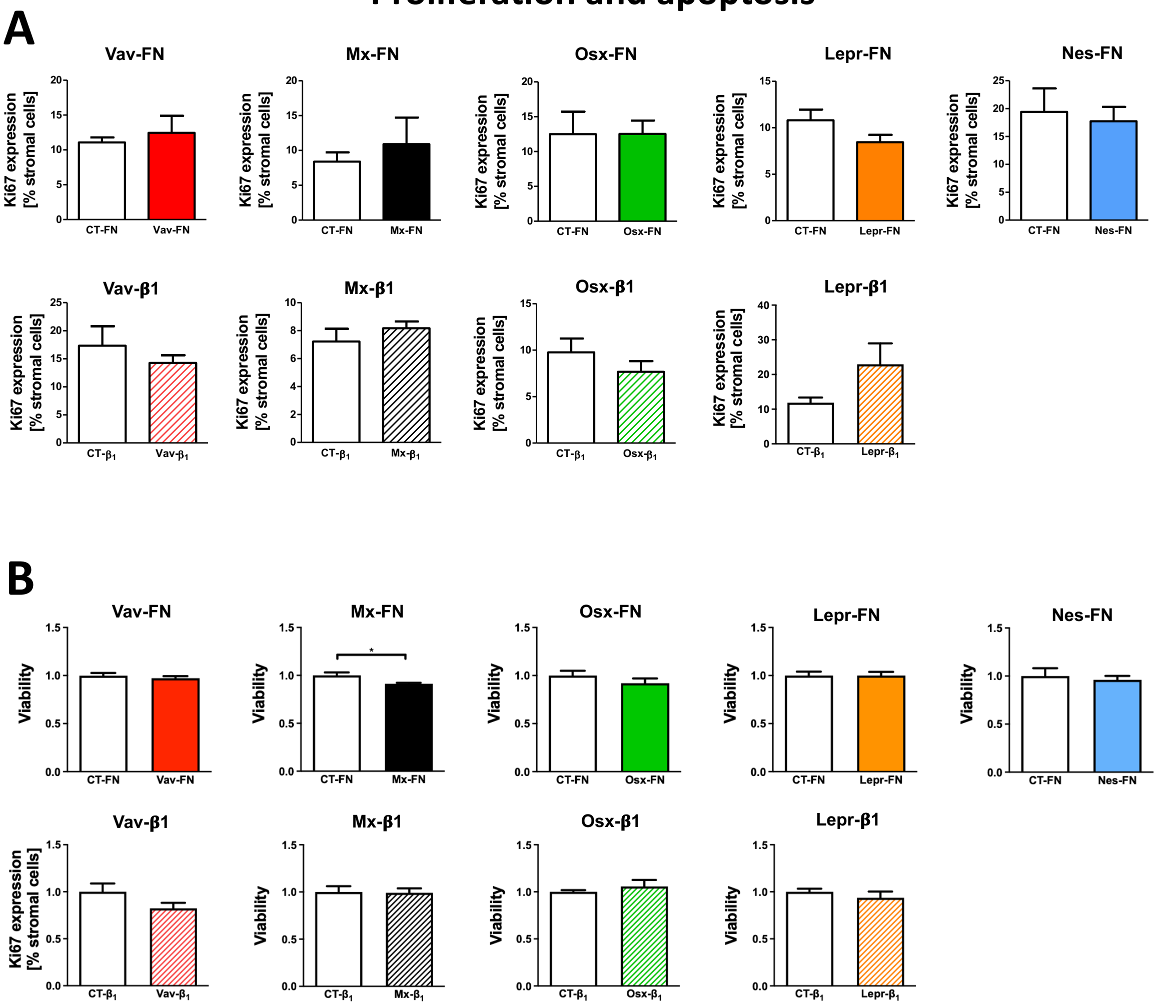

Supplementary Figure 5.
Proliferation and viability of stromal cells in the transgenic mouse models
Freshly isolated bone marrow stromal cells, these cells were isolated from the various mice and cultured for 24 hours, dissociated from the plastic, stained for ki67 as well as propidium iodide, and evaluated by flow cytometry. No differences were detected in any of the models except in Mx-FN, a model in which neither tumor cell homing nor stromal cells were affected.
*In the order of the graphs: N for FN genotypes= 12/12, 4/6, 12/10, 5/4, 5/5. N for integrin $\beta 1$ genotypes: 12/15, 12/11, 8/8, 3/5. Differences between the control (CT) group and the transgenic group were evaluated using student's t-tests. *$p<0.05$.*
To assess proliferation, cells were fixed in 1% PFA and permeabilized using 0.1% triton X. Cells were then blocked in 5% BSA for 15 minutes before staining them with antibodies against Ki67 for 30 minutes. To evaluate viability, propidium iodide was added 5 minutes before measurement of the corresponding sample. The average number of cells from control mice that did not stain with propidium iodide were set at 1 and the viability of the cells from transgenic mice evaluated in relation to their respective controls.

Supplementary Figure 6
No tumor cells

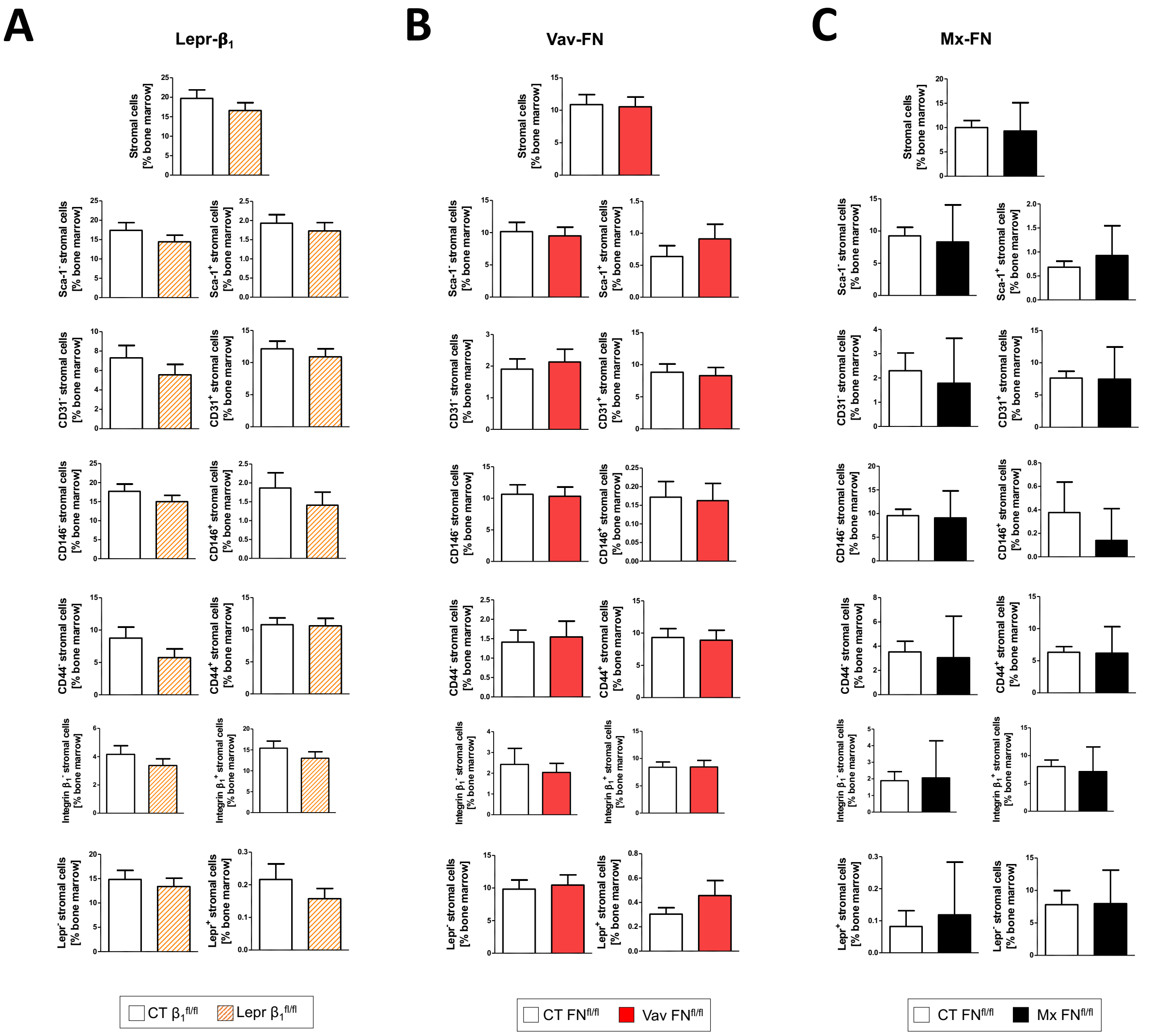

Supplementary Figure 6.

Characterization of stromal cell subpopulations in Lepr-β1 and in two models (Vav-FN and Mx-FN) without change in homing <u>in the absence of tumor cells</u>

In the Lepr-β1 with increased homing as well as in Vav-FN and Mx-FN without changes in homing, the stromal cell subpopulations in the bone marrow remained unaffected as shown for Lepr-β1 (A) (N=43/44), Vav-FN (B) (N=22/21), and Mx-FN (C) (N=22/21). Cells isolated from the bone marrow were stained with a viability marker, CD45 and Ter119 to exclude hematopoietic cells, as well as various stromal markers. Differences between the control (CT) group and the transgenic group were evaluated using student's t-tests. No significant changes were detected. Note that in Lepr-β1 no significant differences for Leptin receptor (Lepr) staining were detected because the data are presented in relation to total bone marrow in contrast to Figure 2F, where cells were evaluated in relation to stromal cells only.

Supplementary Figure 7
No tumor cells

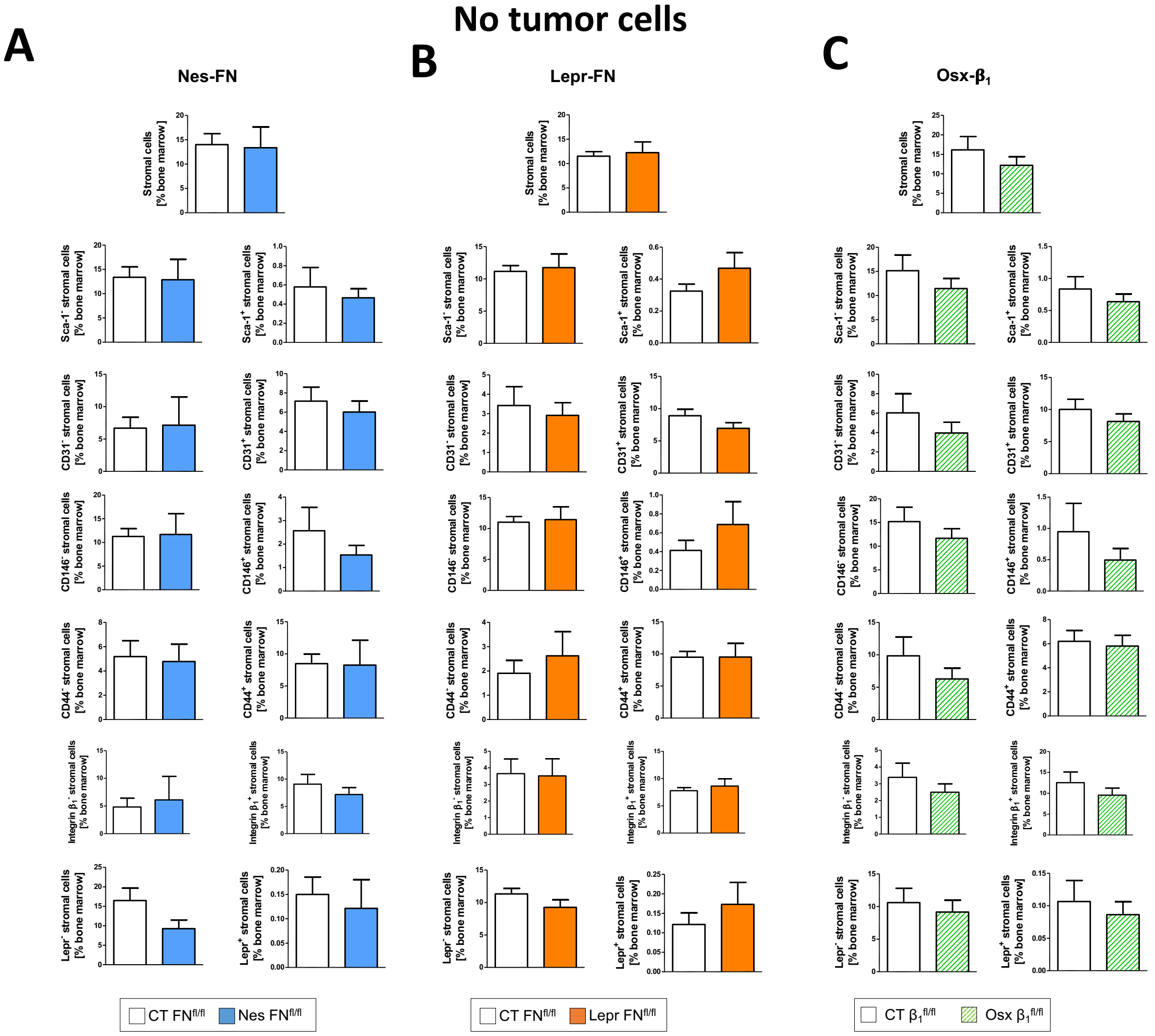

Supplementary Figure 7.

Characterization of stromal cell subpopulations in Nes-FN, Lepr-FN and Osx-β1 <u>in the absence of tumor cells</u>

No changes in total stromal cell or subpopulations in the bone marrow of Nes-FN (A), N= 18/13), Lepr-FN (B) (N= 17/19), or Osx-β1 (C) (N=22/15). In all these models homing of cancer cells was not affected. Cells isolated from the bone marrow were stained with a viability marker, CD45 and Ter119 to exclude hematopoietic cells, as well as various stromal markers. Differences between the control (CT) group and the transgenic group were evaluated using student's t-tests. No significant changes were detected.

Supplementary Figure 8

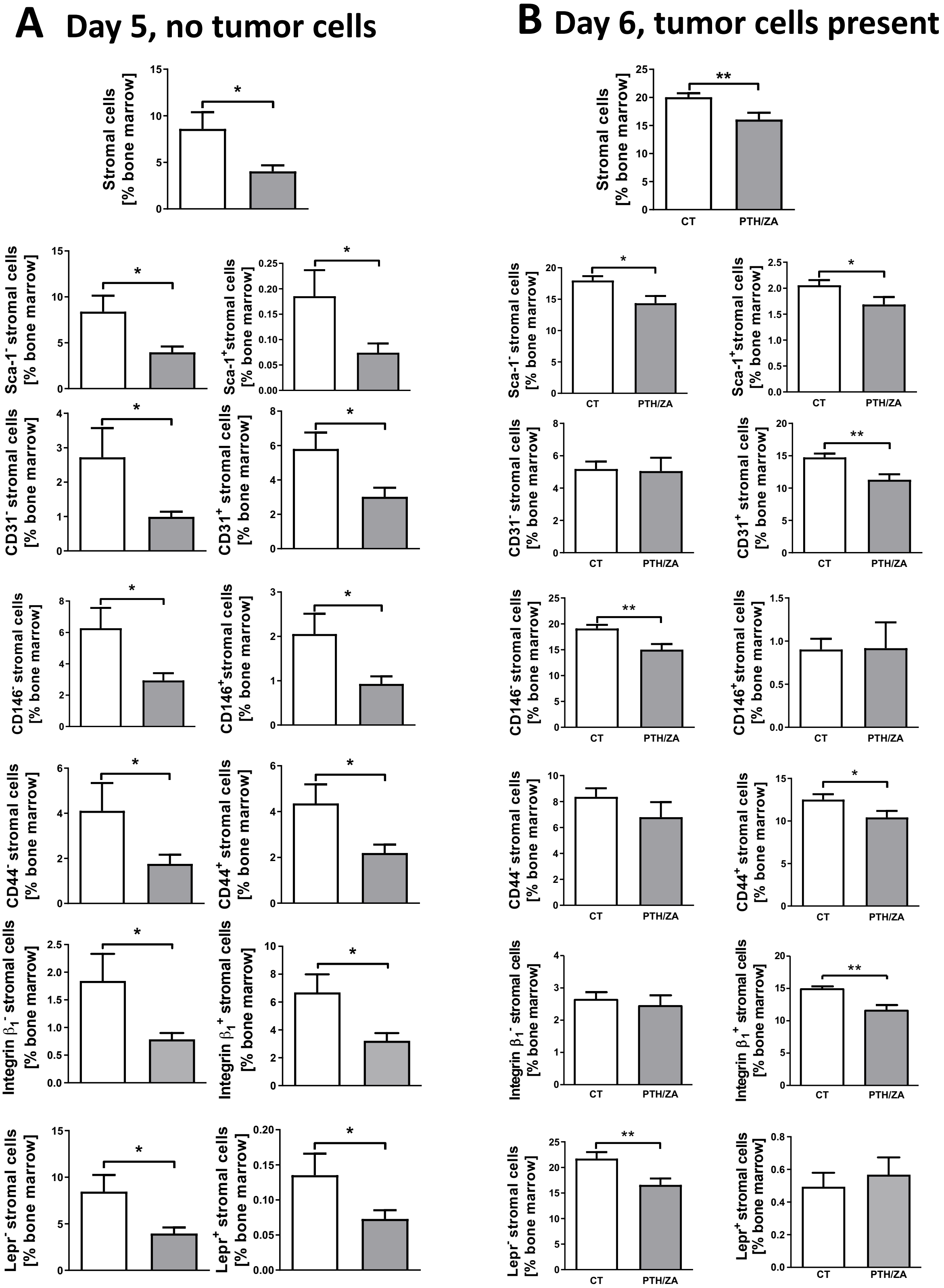

Supplementary Figure 8.
Changes in markers on stromal cells in relation to the total bone marrow in PTH/ZA-treated mice after 5 days in the absence of tumor cell injection (A) or after 6 days from the start of treatment and 24 hours after tumor cell injection (B)

This figure demonstrates the changes in stromal cell markers in relation to the total bone marrow population following treatment with parathyroid hormone (PTH) and zoledronic acid (ZA) in mice. Evaluation of the expression of the stromal markers used in the transgenic mouse models reveals several changes. Percentages were compared using student's t-test. Cells isolated from the bone marrow were stained with a viability marker, CD45 and Ter119 to exclude hematopoietic cells, as well as various stromal markers. N=8/12 ***in A (day 5, no tumor cell injection) and 20/22 in B (day 6, 24 hours after tumor cell injection). Note that the differences in percentages and significances stem from the fact that in A mice were killed on day 5, 24 hours after the last injection of PTH, while in B, mice were killed on day 6, 48 hours after the last injection of PTH.*** *(See Figure 5A for the treatment protocol). Percentages were compared using student's t-test. *p<0.05, **p<0.01.*

Supplementary Figure 9

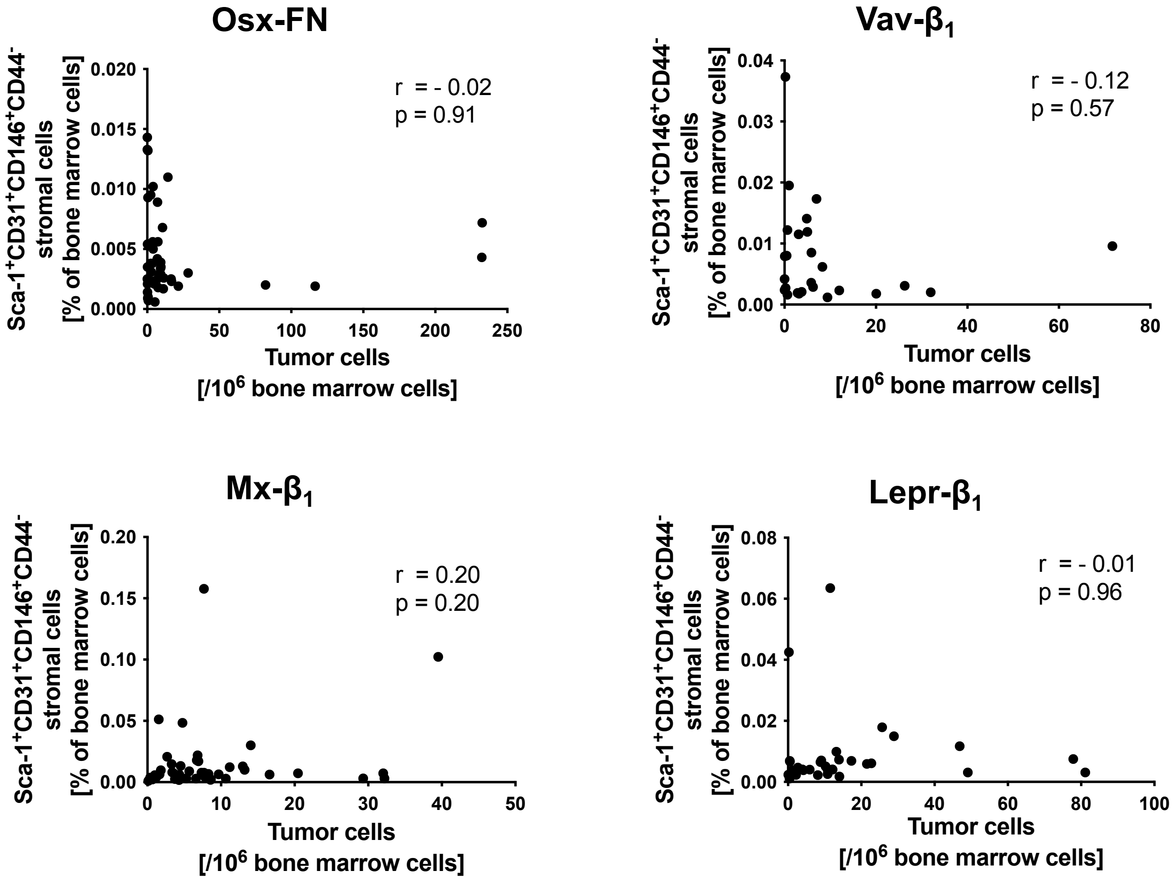

Supplementary Figure 9.

Absence of a relationship between the previously reported stromal cell population and tumor cell homing to the bone marrow in the transgenic mice

Tumor cell homing did not correlate in the four models with increased homing (Osx-FN, Vav-β1, Mx-β1, Lepr-β1) with the subpopulation previously identified in the PTH/ZA model. The genotype is shown above the graphs: Osx-FN (N=40 pairs), Vav-β1 (N=26 pairs), Mx-β1 (N=43 pairs), Lepr-β1 (N=35 pairs). Pearson's correlation was evaluated. None was significant.

Supplementary Figure 10

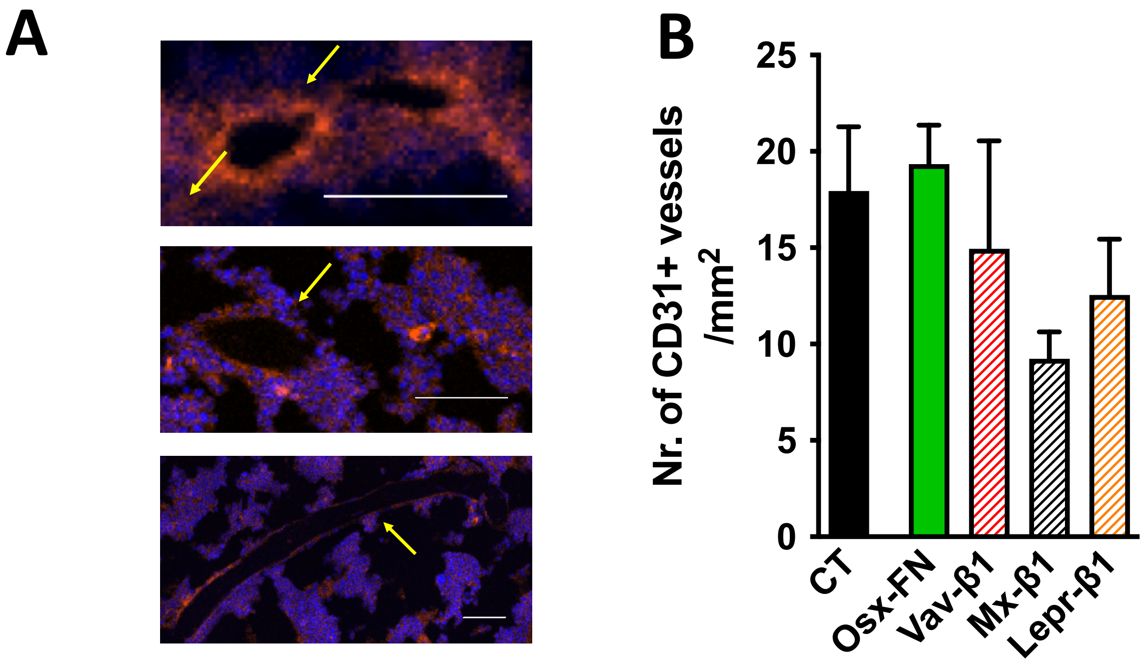

Supplementary Figure 10.
Number of $CD31^+$-vessels in the bone marrow of the genotypes with increased homing of cancer cells. *A. Enlarged pictures to show the small $CD31^+$ vessels (arrow, top picture), mid-size (arrow, middle picture), and large vessels (arrow, lower picture). The bar represents 50 μm. B. The number of $CD31^+$-expressing blood vessels did not differ between the control mice (CT) and the genotypes with increased homing. It should be noted, however, that the sinusoidal vessels tend to exhibit low expression of CD31 in their endothelial cells, limiting the usefulness of this evaluation (Owen-Woods and Kusumbe 2022). N=9/3/3/3/3. Decalcified bones of the various genotypes were sectioned using the Kawamoto method, stained for CD31 and DAPI, pictures made and evaluated starting 150μm below the growth plate and extending 2 mm towards the diaphysis.*

Supplementary Figure 11

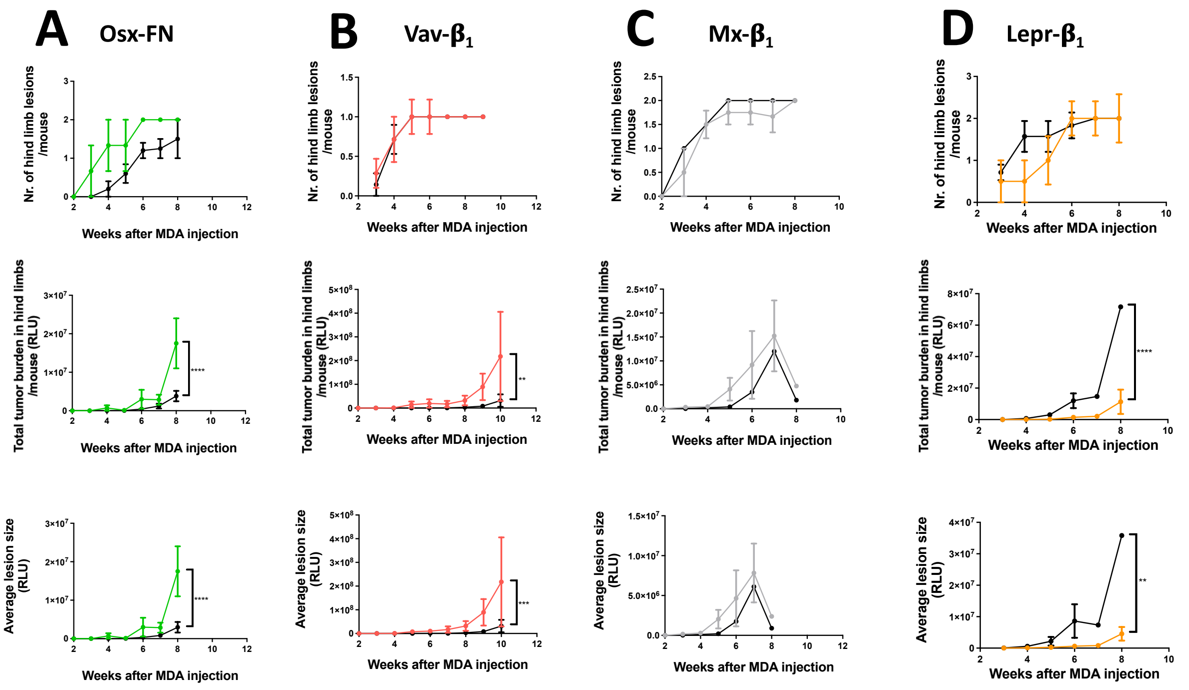

Supplementary Figure 11.

Metastasis in hind limbs of the four models with increased homing

*A-D. Metastatic lesions to the hind limbs are increased in Osx-FN and Vav-β1, but diminished in Lepr-β1. Mice were injected intracardially with tumor cells and growth evaluated weekly using bioluminescence imaging. Only the metastatic lesions that developed in the hind limbs were evaluated. The number of lesions in the hind limbs (upper row), the total tumor burden evaluated by bioluminescence imaging and expressed as relative light units (RLU) is shown in the middle row. The total RLU divided by the number of lesions allows for the quantification of the average lesion size in the hind limbs. These data are shown in the lower row. Comparisons were performed using regression analysis. **$p<0.01$, ***$p<0.001$, ****$p<0.0001$. N= 5/3 (Osx-FN), 7/7 (Vav-β1), 1/4 (Mx-β1), 7/4 (Lepr-β1).*

Supplementary Figure 12

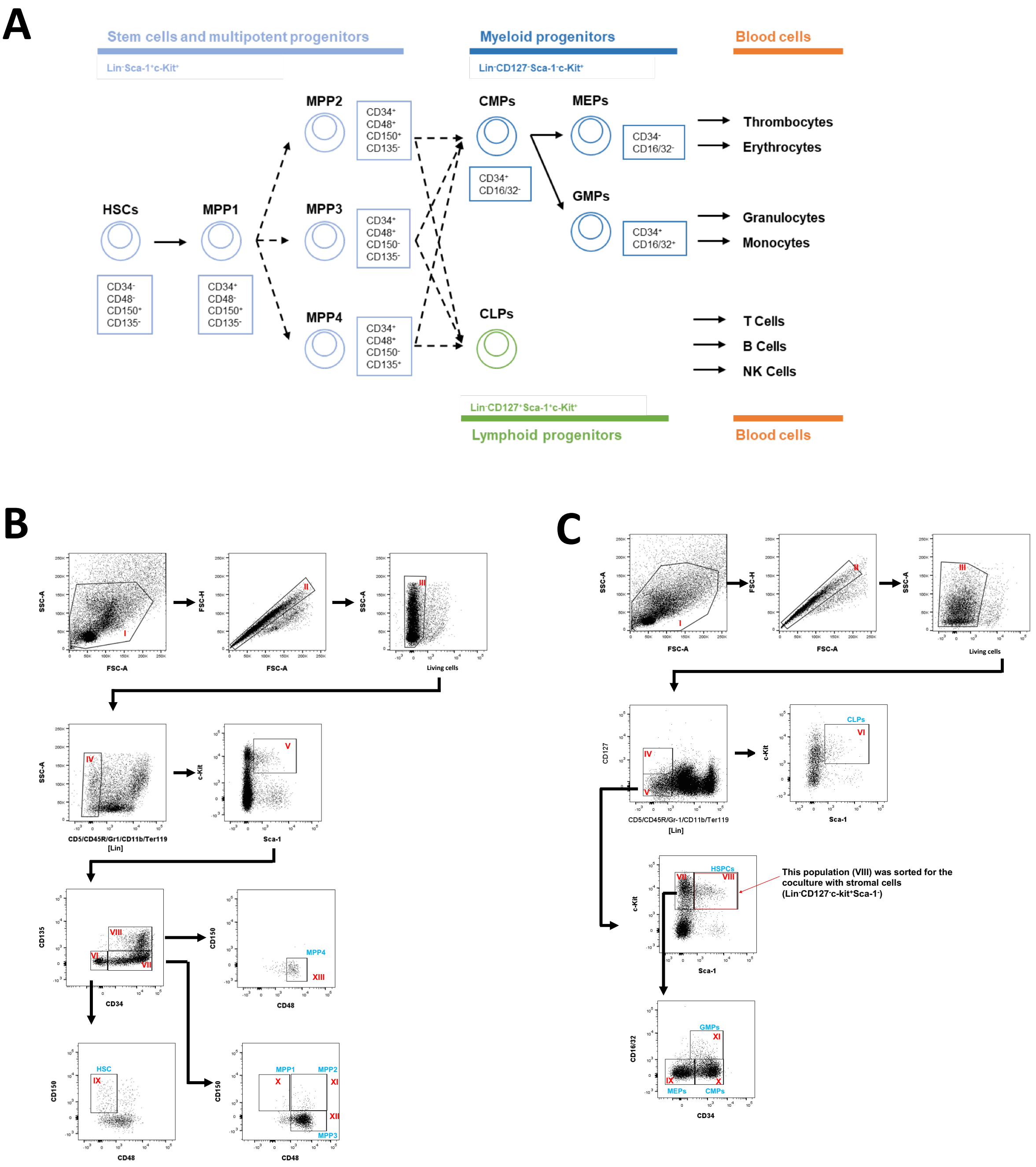

Supplementary Figure 12

Illustration of hematopoiesis progression and gating strategies

A. Schematic of hematopoiesis as evaluated in this work based on (Wilson, Laurenti et al. 2008). B. Flow cytometry gating strategy for evaluation of hematopoietic stem cells (HSCs) and multipotent progenitors (MPPs). C. Flow cytometry gating strategy to sort and evaluate hematopoietic stem and progenitor cells (HSPCs), common myeloid progenitors (CMPs) and common lymphoid progenitors (CLPs), granulocyte-monocyte progenitors (GMPs) and megakaryocyte-erythroid progenitors (MEPs). Based on (Wilson, Laurenti et al. 2008). Sorted HSPCs are surrounded by a red frame. When HSPCs were sorted staining for CD16/32 and CD34 was not needed and therefore not performed.

Supplementary Figure 13

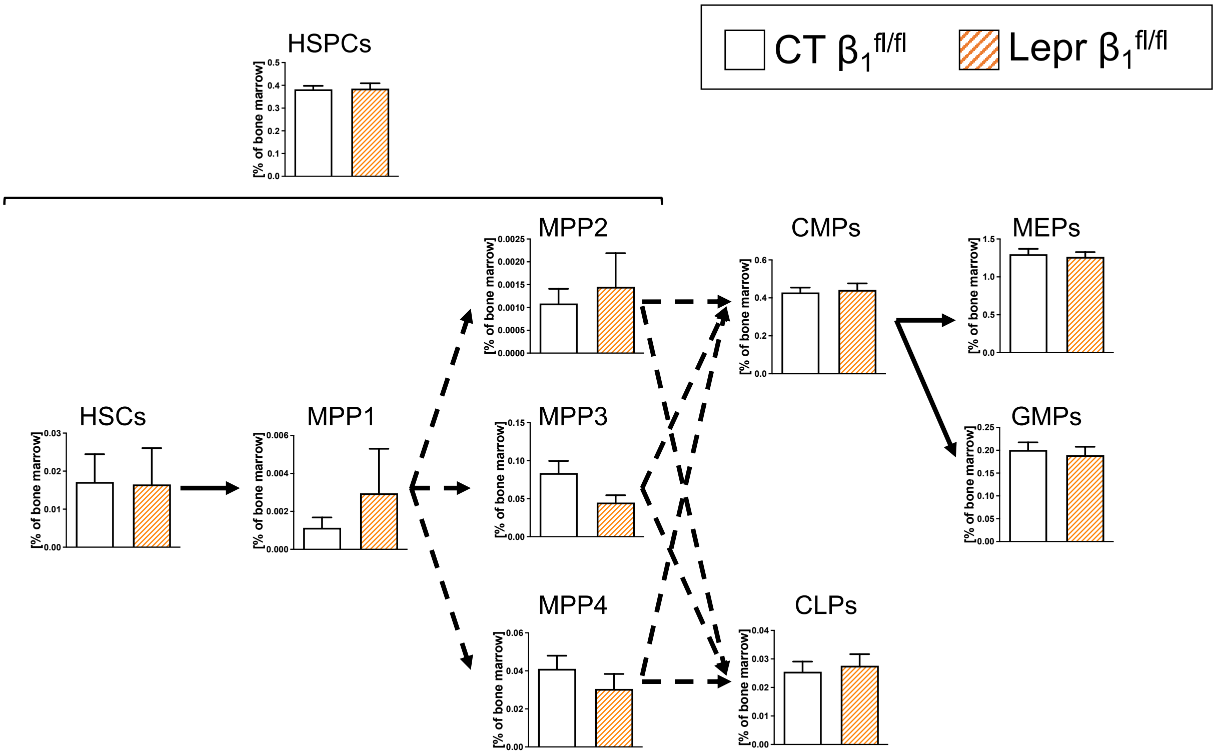

Supplementary Figure 13
Hematopoiesis in Lepr-β1 transgenic mice in vivo
Hematopoiesis in Lepr-β1 mice fails to show any changes in any of the populations evaluated (HSCs, MPPs, HSPCs, CMPs, CLPs, GMPs, and MEPs). Early hematopoietic stem cells (HSCs) and multipotent progenitors (MPPs) were evaluated in the bone marrow separately from evaluation of hematopoietic stem and progenitor cells (HSPCs), common myeloid progenitors (CMPs), common lymphoid progenitors (CLPs), megakaryocyte-erythroid progenitors (MEPs), and granulocyte-monocyte progenitors (GMPs) were evaluated in the bone marrow. N=7/8 for HSCs and MPPs, and 56/57 for HSPC and the rest (Lepr-β1). Data were compared using student's t-tests. No significant changes were detected.

Supplementary Figure 14

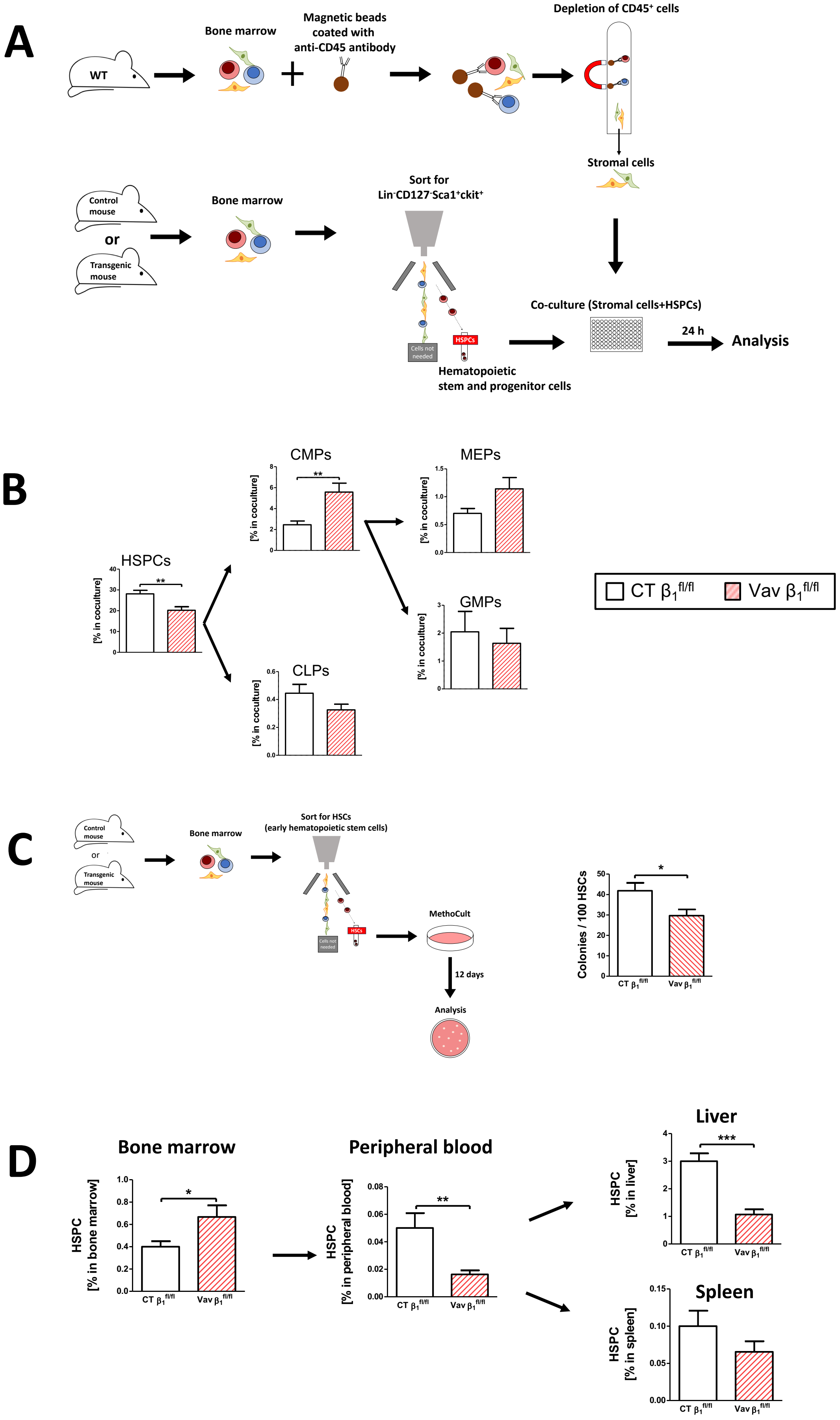

Supplementary Figure 14
Changes in hematopoiesis attributable to HSPCs from Vav-β1 mice
*A. Scheme for evaluation of the role of HSPCs from transgenic mice. HSPCs sorted from transgenic mice are co-cultured with stromal cells isolated from wildtype mice and hematopoiesis evaluated 24 hours later. B. HSPCs from Vav-β1 mice diminished when cocultured with wildtype stromal cells. N=10/11. C. Culture of sorted Vav-β1 HSCs (Lin$^-$ c-kit$^+$Sca-1$^+$CD34$^-$CD135$^-$CD48$^-$CD150$^+$) and differentiation over 12 days in specialized media (MethoCult) confirmed a decrease in the ability of these cells to differentiate. 100 cells were added to each well. N=12/11. D. In view of the increase in HSPCs in vivo we wondered whether there was an increase in retention of HSPCs in the bone marrow. This seemed to be the case, because HSPCs were diminished in the peripheral blood as well as in the liver. In the spleen, the difference was not statistically significant. HSPCs (hematopoietic stem and progenitor cells), CMPs (common myeloid progenitors) and CLPs (common lymphoid progenitors), the GMPs (granulocyte-monocyte progenitors) and MEPs (megakaryocyte-erythroid progenitors) were examined by flow cytometry. N=9/9. Data were compared using student's t-tests. *p<0.05, **p<0.01, ***p<0.005.*

Supplementary Table 1. Evaluation of cutoffs in stromal CD31+ integrin β1+ Sca1- cells in relation with tumor cells detected in the bone marrow. *At any specific cutoff the mean number of tumor cells in the bone marrow for the mice with less cells of the stromal subpopulation (a lower percentage) was higher than for mice with a higher percentage of the stromal subpopulation (more than the cutoff). A cutoff between 2 and 5% of the stromal subpopulation resulted in significant separation in mean tumor cell numbers in the bone marrow in the three models. Since all mice of the genotype Vav-β1 had less than 4% of the stromal cell subpopulation, the row including this cutoff is highlighted. In all models, a 4% cutoff resulted in significant differences. Mean numbers of tumor cells were evaluated by t-test. *p<0.05, **p<0.01. The empty cells do not show significant differences in tumor cell numbers.*

**Cut-off** for % of $CD31^+$ Integrin $\beta1^+$ Sca-1^- cells	**Osx-FN**			**Vav-β1**			**Mx-β1**		
	Nr. of cancer cells (mean/10^6 bone marrow cells) ≤ cutoff vs. > cutoff	p	Replicates (≤ vs. >)	Nr. of cancer cells (mean/10^6 bone marrow cells) ≤ cutoff vs. > cutoff	p	Replicates (≤ vs. >)	Nr. of cancer cells (mean/10^6 bone marrow cells) ≤ cutoff vs. > cutoff	p	Replicates (≤ vs. >)
2%	62 vs. 5	**	12/28	27 vs. 5	**	4/22	22 vs. 8	**	3/40
3%	53 vs. 5	**	14/26	22 vs. 5	*	5/21	17 vs. 7	**	8/35
4%	47 vs. 5	*	16/24	19 vs. 5	*	7/19	14 vs. 6	**	15/28
5%	44 vs. 5	*	17/23	19 vs. 4	*	9/17	13 vs. 6	*	18/25
6%				19 vs. 4	*	9/17	13 vs. 6	*	18/25
7%				17 vs. 4	*	10/16	12 vs. 6	*	21/22
8%				16 vs. 4	*	11/15			

Supplementary Table 2. Expression of molecules involved in adhesion and transmigration of cancer cells to endothelial cells. *References in italics discuss adhesion effects, while those in standard font relate to transmigration. Blue cells mark statistically significant differences between transgenic cells and littermate controls. #p>0.05 and <0.10, *p<0.05, **p<0.01.*

		Osx-FN (11/11)			Vav-β1 (12/13)			Mx-β1 (4/4)			Lepr-β1 (4/5)		
		CT	cKO	p	CT	cKO	p	CT	cKO	p	CT	cKO	p
**CD31+** (Ferrero, Elena et al. 1995)	**% to all stromal cells**	51.8±3.5	37.8±3.7	*	56.4±6.7	33.8±5.2	*	73.6±5.3	49.2±9.5	*	23.9±4.7	20.4±1.3	
	**MFI in CD31+ cells**	329±14.9	286±18	#	500±8	424±25	*	1410±126	1583±181		695±48	564±22	*
**ICAM-1 (CD54)** *(Strell and Entschladen 2008, Sökeland and Schumacher 2019)*	**% to all stromal cells**	25.8±1.4	23.1±2.5		20.2±1.8	12.1±1.3	**	27.1±3.2	24.4±3.0		27.8±3.6	25.0±1.7	
	**% to CD31+ cells**	40.0±1.9	44.2±1.8		28.3±1.4	26.4±1.6		32.0±4.7	31.9±4.6		55.2±3.8	51.7±3.9	
	**MFI in CD31+ICAM-1+**	868±32	915±35		1361±44	1399±55		178±2	186±4		242±15	248±10	
**VCAM-1 (CD106)** *(Strell and Entschladen 2008)*	**% to all stromal cells**	5.4±0.6	4.1±0.6		6.2±0.7	5.4±1.1		23.1±5.5	22.0±3.0		24.1±2.0	26.3±3.2	
	**% to CD31+ cells**	3.1±0.5	1.1±0.7		3.8±0.2	3.2±0.4		23.9±5.6	27.7±4.5		29.7±2.6	31.9±3.2	
	**MFI in CD31+VCAM-1+**	1228±42	1441±95	#	1170±52	1028±37		604±37	637±51		1057±201	1316±135	
**VE-Cadherin (CD144)** (Matsuyoshi, Toda et al. 1997, Frye, Dierkes et al. 2015)	**% to all stromal cells**	0.15±0.02	0.18±0.03		0.30±0.04	0.32±0.03		0.58±0.16	0.49±0.20		0.30±0.05	0.46±0.20	
	**% to CD31+ cells**	0.16±0.01	0.35±0.08	*	0.30±0.05	0.52±0.08	*	0.32±0.08	0.19±0.04		1.04±0.33	1.38±0.49	
	**MFI in CD31+VE-cadherin+**	3.36±0.02	3.30±0.04		297±9	452±70		483±115	691±256		1290±68	1125±111	
**ESAM** (Duong, Nottebaum et al. 2020)	**% to all stromal cells**	0.16±0.02	0.15±0.02		0.18±0.02	0.15±0.01		2.08±0.25	3.95±0.96		3.3±0.5	4.3±0.8	
	**% to CD31+ cells**	0.27±0.05	0.38±0.06		0.30±0.05	0.46±0.06	*	1.46±0.15	3.03±1.13		13.9±2.7	13.7±4.8	
	**MFI in CD31+ESAM+**	1130±24	1316±64	*	1507±111	1422±72		499±51	440±55		561±51	602±54	
**JAM-A** (Mandell, Holley et al. 2006)	**% to all stromal cells**	1.0±0.3	0.8±0.1		0.83±0.14	0.84±0.12		1.9±0.6	5.1±1.8		2.7±0.8	3.5±1.1	
	**% to CD31+ cells**	2.0±0.8	2.1±0.3		1.3±0.2	2.3±0.3	*	1.5±0.2	3.0±1.1		13.1±4.1	16.3±4.5	
	**MFI in CD31+JAM-A+**	910±64	1066±85		1256±96	1565±73	*	2056±420	3011±333		1548±82	1615±33	

Supplementary Table 3. *Cytokines affected in the various genotypes. Stromal cells were isolated and cultured for 24 hours. Then two cytokine arrays were run on the conditioned media. Blue cells mark statistically significant differences between transgenic cells and littermate controls. *p<0.05; **p<0.01; ***p<0.005; #p>0.05 and <0.10.*

	Osx-FN			Vav-β1			Mx-β1			Lepr-β1		
	CT	cKO	N	CT	cKO	N	CT	cKO	N	CT	cKO	N
**IL-3**	1.49±0.04	1.47±0.08	6/5	1.36±0.03	1.37±0.02	3/3	1.18±0.11	1.35±0.16	12/11 #	0.86±0.10	0.93±0.11	9/14
**IL-4**	0.87±0.06	0.98±0.07	3/4	1.07±0.22	1.17±0.17	3/3	0.85±0.24	1.17±0.42	10/12	0.05±0.02	0.36±0.14	8/13 #
**IL-5**	0.37±0.11	0.46±0.13	6/5	0.57±0.14	0.59±0.12	3/3	0.37±0.07	1.03±0.3	15/6 **	0.26±0.07	0.31±0.06	6/13
**IL-6**	37.08±8.42	28.69±6.46	5/5	18.54±7.62	13.77±1.04	3/3	7.84±1.44	29.02±10.86	12/12 #	55.46±7.77	10.88±4.71	6/12 ***
**IL-6 (Cy)**	13.42±3.27	10.81±1.51	3/4	7.73±4.15	3.99±0.77	3/3	5.30±1.38	11.30±3.70	7/8	2.10±0.55	0.77±0.28	8/12 *
**IL-10**	38.50±4.81	36.13±1.87	3/3	17.63±1.73	14.52±0.59	3/3	19.52±2.24	17.32±1.64	7/11	1.02-0.37	1.11±0.18	6/11
**IL-15**	3.94±0.54	3.68±0.95	6/5	3.20±0.68	2.47±0.44	3/3	7.22±4.10	9.99±4.48	6/9	8.16±4.08	7.71±2.39	4/8
**IL-34**	9.11±1.29	7.81±3.38	6/4	12.78±7.95	6.15±1.31	2/3	5.74±1.84	14.01±3.94	8/9	9.25±2.93	14.48-3.09	5/11
**M-CSF**	1.84±0.64	3.32±0.80	5/4	-	-	-	3.04±1.05	9.27±5.19	9/6	3.24±1.15	4.76±1.05	8/10
**GM-CSF**	2.31±0.11	1.91±0.36	6/5	2.05±0.63	1.22±0.36	3/3	1.90±0.31	1.9±0.26	11/11	1.44±0.27	2.08±0.21	9/11 #
**GM-CSF (CR)**	1.54±0.23	1.77±0.18	3/4	1.71±0.23	1.33±0.04	3/3	1.055±0.29	1.10±0.36	12/12	0.23±0.06	0.27±0.04	9/13
**EPO**	3.36±0.02	3.30±0.04	6/5	3.19±0.02	3.34±0.04	3/3 *	2.00±0.36	2.57±0.54	12/12	0.81±0.02	0.87±0.03	9/14
**TPO**	1.38±0.19	1.06±0.15	6/5	1.21±0.44	1.32±0.04	3/3	2.17±0.35	1.98-0.34	10/11	2.46±0.95	2.46±0.37	7/12
**LIF**	2.44±0.22	2.20±0.13	6/5	1.78±0.11	2.06±0.11	3/3	1.71±0.16	1.83±0.2	12/11	1.18±0.19	1.21±0.08	9/14
**SCF**	7.17±0.35	6.88±0.61	6/5	6.03±0.47	7.02±0.22	3/3	4.24±0.99	7.92±1.36	8/8 *	7.95±3.56	6.79±2.32	3/7
**IFN-α**	-	-	-	1.69±0.18	1.48±0.05	3/3	0.90±0.23	0.85±0.21	12/12	0.18±0.02	0.21±0.03	9/15
**IFN-γ**	2.44±0.25	2.13±0.18	3/4	1.83±0.11	1.70±0.19	3/3	29.17±8.94	25.33±6.84	12/12	0.09±0.02	0.06±0.02	7/11
**TNF-α**	-	-	-	12.71±3.18	13.84±2.45	3/3	6.20±1.84	6.70±2.37	8/7	0.59±0.19	0.51±0.11	3/10
**TGF-β**	2.22±0.27	2.02±0.48	6/5	1.40±0.01	1.59±0.38	3/3	8.02±2.28	7.72±2.38	10/9	11.23±1.31	15.54±1.13	9/13 *
**CXCL9**	4.27±0.23	3.86±0.40	3/4	3.33±0.38	3.25±0.11	3/3	1.94±0.45	1.98±0.48	11/11	0.64±0.25	0.45±0.12	9/6
**CXCL10**	-	-	-	125.60±32.37	149.50±41.84	3/2	26.67±11.15	23.82±9.56	10/11	8.21±4.41	2.17±0.32	8/15 #
**CXCL12**	82.40±9.70	72.35±1.77	6/4	56.45±3.63	56.45±2.36	3/3	67.15±6.28	58.98±2.82	12/10	70.90±8.76	65.18±5.29	9/14
**CCL2**	13.30±7.32	7.04±0.93	3/4	25.04±18.36	7.40±1.44	3/3	16.53 ±6.82	13.01±4.74	10/11	1.91±0.52	1.84±0.43	7/14
**CCL3**	30.44±3.97	33.14±5.46	2/2	7.03±1.36	6.12±0.74	3/3	4.48±1.04	6.88±1.95	9/8	4.62±0.54	1.29±0.38	3/13 **
**CCL4**	10.16±0	23.68±14.55	1/3	12.27±2.53	16.88	3/1	10.53±2.92	9.28±2.55	12/12	4.95±1.61	2.84±0.61	6/15
**VEGF**	-	-	-	35.13	35.51±5.63	1/2	0.46±0.22	8.84±5.70	2/7	0.54±0.16	1.28±0.50	6/13

IL: Interleukin; M-CSF: Macrophage-colony stimulating factor; GM-CSF: Granulocyte macrophage-CSF; Epo: Erythropoietin; TPO: Thrombopoietin; LIF: Leukemia inhibitory factor; SCF: stem cell factor, ligand for c-kit receptor; IFN: Interferon; TNF-α: Tumor necrosis factor-α; TGF-β: Transforming growth factor-β; CXCL: Chemokine (C-X-C motif) ligand; CCL: C-C motif chemokine ligand; VEGF: vascular endothelial growth factor.
